# Electrode Droplet Microarray (eDMA): An Impedance Platform for Label‐Free Parallel Monitoring of Cellular Drug Response in Nanoliter Droplets

**DOI:** 10.1002/adhm.202402046

**Published:** 2024-10-15

**Authors:** Meijun Zhou, Joaquin E. Urrutia Gomez, Nikolaj K. Mandsberg, Sida Liu, Sabine Schmidt, Matthias Meier, Pavel A. Levkin, Heinz‐Georg Jahnke, Anna Popova

**Affiliations:** ^1^ Institute of Biological and Chemical Systems – Functional Molecular Systems (IBCS‐FMS) Karlsruhe Institute of Technology Kaiserstrasse 12 76131 Karlsruhe Germany; ^2^ Karlsruhe Institute of Technology (KIT) Institute of Automation and Applied Informatics (IAI) Eggenstein‐Leopoldshafen 76344 Karlsruhe Germany; ^3^ Centre for Biotechnology and Biomedicine Biochemical Cell Technology Leipzig University Deutscher Platz 5 D‐04103 Leipzig Germany; ^4^ Institute of Organic Chemistry Karlsruhe Institute of Technology Kaiserstrasse 12 76131 Karlsruhe Germany

**Keywords:** drug screening, electrode droplet microarray, impedance, label‐free

## Abstract

Label‐free real‐time monitoring of cellular behavior using impedance spectroscopy is important for drug development and toxicological assessments. Parallelization and miniaturization of such experiments are essential for increasing throughput and enabling experiments with low abundant stem or primary cells. Traditional methods are not miniaturized and require large volumes of reagents and number of cells, limiting their suitability for cost effective high‐throughput screening of cells of limited availability. Here, the fabrication, optimization, and application of a bioelectrical signaling monitoring system – electrode droplet microarray (eDMA) are demonstrated. The eDMA platform is based on preparation of a hydrophilic‐superhydrophobic patterns covering an array of individually addressable microelectrodes, which confines cells to individual microelectrodes, allowing for parallel, real‐time, and label‐free detection of cellular responses to drug treatments in nanoliter droplets. The real‐time monitoring of cytotoxic effect of an anticancer drug is demonstrated over 48 h with real‐time calculation of the half‐inhibitory concentration (IC50) values through impedance spectroscopy. This demonstrates eDMA's ability to dynamically assess responses to various drugs in parallel at any given time point, which is crucial for functional personalized oncology. Specifically, the platform can be employed for monitoring anticancer drug toxicity using limited patient samples, where the miniaturization provided by eDMA is essential.

## Graphical abstract

This study combines hydrophilic‐superhydrophobic patterns with microelectrodes to miniaturize cellular bioelectrical signal detection to nanoliter volumes. This method enables precise monitoring of cellular responses to drug treatments within nanoliter droplets formed on the hydrophilic spots containing the electrodes.

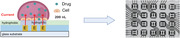

## 1 Introduction

Assessing the viability of cancer cells in in‐vitro culture is fundamental to preclinical evaluation of anticancer drugs,^[^ ^1^ ^]^ functional personalized oncology, and fundamental biological research. Popular methods for determining viability include the 3‐(4,5‐dimethylthiazol‐2‐yl)‐2,5‐diphenyltetrazolium bromide (MTT) assay, ATP luminescence‐based assays, fluorescence viability dyes used with microscopy or flow cytometry, and apoptosis assays. These methods help quantify the sensitivity or resistance of cancer cells to a given drug by extracting indicators such as half inhibitory concentration (IC50), resistance index (RI), cell proliferation index, cell growth curve, and apoptosis index.^[^ ^2^, ^3^ ^]^ These assays are well established, easy to use, and compatible with high throughput screening workflows. However, these approaches require labeling and are mostly end‐point assays without the possibility to do real‐time monitoring of cell responses. Labeling itself can directly impact cell behavior and viability, masking subtle nuances in biological drug response. For instance, the commonly used MTT reagents are known to be toxic.^[^ ^4^, ^5^ ^]^ While some fluorescent viability assays have been developed for real‐time monitoring of cell viability using microscopy for live cell imaging,^[^ ^6^ ^]^ fluorescent dyes are prone to photobleaching, leading to irreproducible results and cell harm during prolonged imaging.^[^ ^7^ ^]^ Cancer cell lines, especially primary patient‐derived cells, may exhibit nuanced differences in their response to drugs, with the timing of drug exposure often playing a crucial role.^[^ ^8^ ^]^ Therefore, the development of label‐free platforms for high throughput real‐time monitoring of cell viability is essential for research and drug development in oncology.

Electrochemical Impedance Spectroscopy (EIS) is a powerful analytical technique that is used for real‐time monitoring of cell behavior.^[^ ^9^ ^]^ It involves applying a small amplitude alternating current (AC) to an electrode pair in solution and measuring the resulting voltage response. The calculated impedance as a function of AC frequency provides detailed information about various processes occurring near the electrode´s surface. As cells attach and spread on the electrodes, the impedance changes.^[^ ^10^ ^]^ EIS is a label‐free and non‐invasive method that enables monitoring the impedance over time giving real‐time insight into cell attachment kinetics and behavior.^[^ ^11^, ^12^ ^]^

Microelectrode array (MEA) technology enables non‐invasive and real‐time monitoring of cell density, supporting various formats, including a single MEA chip, as well as 6, 24, 48, 96, and 384 well plates. In these systems each well is equipped with microelectrodes to detect changes in cellular electrical signals, handling volumes ranging from 100 µL to 3 mL. This technology is utilized for various applications, for example for the dynamic assessment of neuronal activity and mechanosensitive channel functionality in response to drugs,^[^ ^13^ ^]^ for investigating the developmental neurotoxicity of various compounds by using human‐induced pluripotent stem cell‐derived neurons and astroglia,^[^ ^14^ ^]^ or for monitoring for chemotherapy drug‐induced peripheral neurotoxicity.^[^ ^15^ ^]^

The miniaturization of cell assays is important for saving the reagents and valuable cells, such as scarce primary cells or challenging‐to‐culture stem cells. Miniaturized platforms for bioelectrical signal detection that combine impedance with microfluidic‐based platforms can be used for the detection and analysis of cells,^[^ ^16^, ^17^, ^18^, ^19^ ^]^ proteins,^[^ ^20^, ^21^, ^22^, ^23^ ^]^ bacteria,^[^ ^24^ ^]^ DNA,^[^ ^25^, ^26^ ^]^ and hormones.^[^ ^27^ ^]^ In addition, cells can be cultured and exposed to drugs for monitoring in vitro drug responses inside microfluidic channels in these platforms. Some examples of applications demonstrated in such platforms include, assessment of the dose‐dependent viability of anti‐schistosomal drugs,^[^ ^28^ ^]^ monitoring the cytotoxic effect of anticancer drugs,^[^ ^29^ ^]^ and assessment of epithelial barrier function under treatment with corticosteroids.^[^ ^30^ ^]^

**Figure 1:**
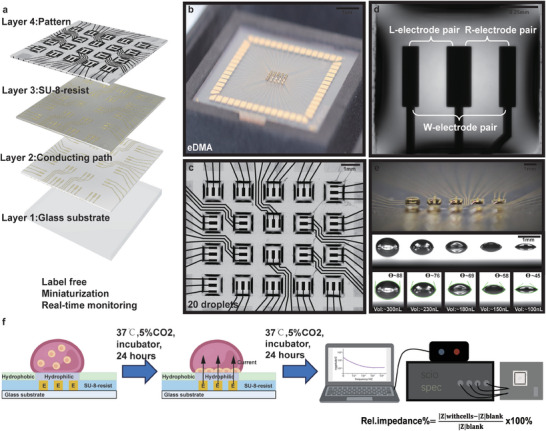
Schematic concept of the eDMA platform. a) Schematic representation of the eDMA structure composed of four layers: 1. glass substrate, 2. gold electrodes, 3. SU‐8‐resist and 4. nanoparticle‐based coating with a hydrophilic‐superhydrophobic pattern. b–c) Photographs of eDMA, showing the electrical circuit and the 5 × 4 array of 200 nL droplets formed on square 1 mm^2^ hydrophilic spots each containing three gold electrodes. d) A photograph of a single 200 nL droplet on eDMA, containing three electrode pairs: L‐electrode pair consisting of L and W electrode, R‐electrode pair consisting of R and W electrode and W‐electrode pair consisting of L and R electrodes. e) A photograph of the eDMA containing indicated volumes of water droplets. The lower panel shows the values of apparent water contact angle (θ). f) The workflow for real‐time monitoring of cell density on eDMA.

In the current study, EIS was combined for the first time with an open array of droplets on a planar surface using the Droplet Microarray (DMA) platform. The DMA platform has been developed in our laboratory and consists of an array of hydrophilic spots surrounded by superhydrophobic borders. Nanoliter‐sized droplets (50–300 nL) can be stably confined on hydrophilic spots, serving as nano‐wells for cell culture and drug treatment.^[^ ^31^ ^]^ Non‐contact liquid dispensers can be applied to dispense cell suspensions and reagents onto hydrophilic spots or already formed droplets on the DMA chips. Overall, the DMA platform is a cost‐effective method for biological and chemical assays, including phenotypic assessment and gene expression analysis,^[^ ^32^ ^]^ high‐throughput formation of miniaturized cocultures,^[^ ^33^ ^]^ high‐throughput screening for antimicrobial compounds^[^ ^34^ ^]^ and pluripotency maintenance proteins,^[^ ^35^ ^]^ screening of embryoid body,^[^ ^36^ ^]^ and stem cells,^[^ ^37^, ^38^, ^39^ ^]^ miniaturized drug sensitivity and resistance test^[^ ^40^, ^41^, ^42^ ^]^ and high‐throughput drug synthesis and screening.^[^ ^43^ ^]^ Here, we established a novel technique termed Electrode Droplet Microarray (eDMA), by combining an array of microelectrodes with the hydrophilic‐superhydrophobic pattern. eDMA enables label‐free and real‐time monitoring of live cells in nanoliter droplets by the EIS (**Figure** **1**). For the first time, we have demonstrated the possibility of continuous monitoring of impedance signal in nanolitre droplets over 48 h on eDMA platform. We have successfully validated eDMA for impedance measurement, showcasing its capability for parallel, real‐time monitoring of both cell growth and drug response within 200 nL droplets. Thus, the eDMA platform holds a promise for enabling miniaturized, parallel, label‐free, and real‐time monitoring of cellular drug responses in nanoliter formats.

## 2 Results and Discussions

### 2.1 Concept of eDMA

The Electrode Droplet Microarray (eDMA) platform comprises four layers (Figure 1a; Figure S1c, Supporting Information): 1. Glass substrate, 2. Conducting paths (gold electrodes), which are printed on cleaned glass surface according to a well‐established procedure (see Experimental Section).^[^ ^44^ ^]^ 3. SU‐8‐resist, which is a negative photoresist used to create precise microstructures. It is patterned through UV exposure and development, revealing the underlying gold electrodes in defined areas where the contact with cells is desired (see Experimental Section).^[^ ^44^ ^]^ 4. Silica nanoparticle coating patterned with hydrophilic and superhydrophobic regions,^[^ ^31^, ^45^ ^]^ which is then chemically modified to create hydrophilic spots on 3 working electrodes and superhydrophobic background by using a photomask.

For this study, we fabricated an eDMA that contained an array of 5 × 4 square hydrophilic spots (in total 20 spots), each 1 mm^2^ in size (Figure 1b,c; Figure S1a, Supporting Information), which covered three working electrodes (2 small electrodes: 0.4 mm × 0.1 mm and 1 big electrode: 0.4 mm × 0.15 mm) (Figure S1b, Supporting Information). Together, the three electrodes formed three electrode pairs for the eDMA: L, R, W (Figure 1d; Figure S1a,b, Supporting Information). The hydrophilic‐superhydrophobic patterning allowed for the precise formation of stable aqueous droplets ranging from 100 to 300 nL on the designated working electrodes (Figure 1e).

Figure 1f depicts the workflow for real‐time monitoring of cell growth and drug response on the eDMA. First, cells were dispensed onto the hydrophilic spots of eDMA using non‐contact low volume dispenser. The eDMA was then inserted into the impedance spectroscopy system placed in a cell culture incubator, where the changes in cell density could be monitored in real time. For this, alternating current (AC) in frequencies ranging from 500 Hz to 5 MHz was applied to the electrodes and impedance amplitude Z (Ohm (Ω)) was measured. Impedance amplitude is influenced by the presence of cells close to the electrode´s surface in a way that the greater the number of cells present, the higher the impedance amplitude. In order to be able to distinguish the impedance signal of cells from that of the medium without cells, we have used relative impedance, which is calculated as: ((Z_cells_ – Z_blank_)/Z_blank_) × 100% (Figure 1f).

### 2.2 Impedance Spectroscopy‐Based Characterization of eDMA

**Figure 2:**
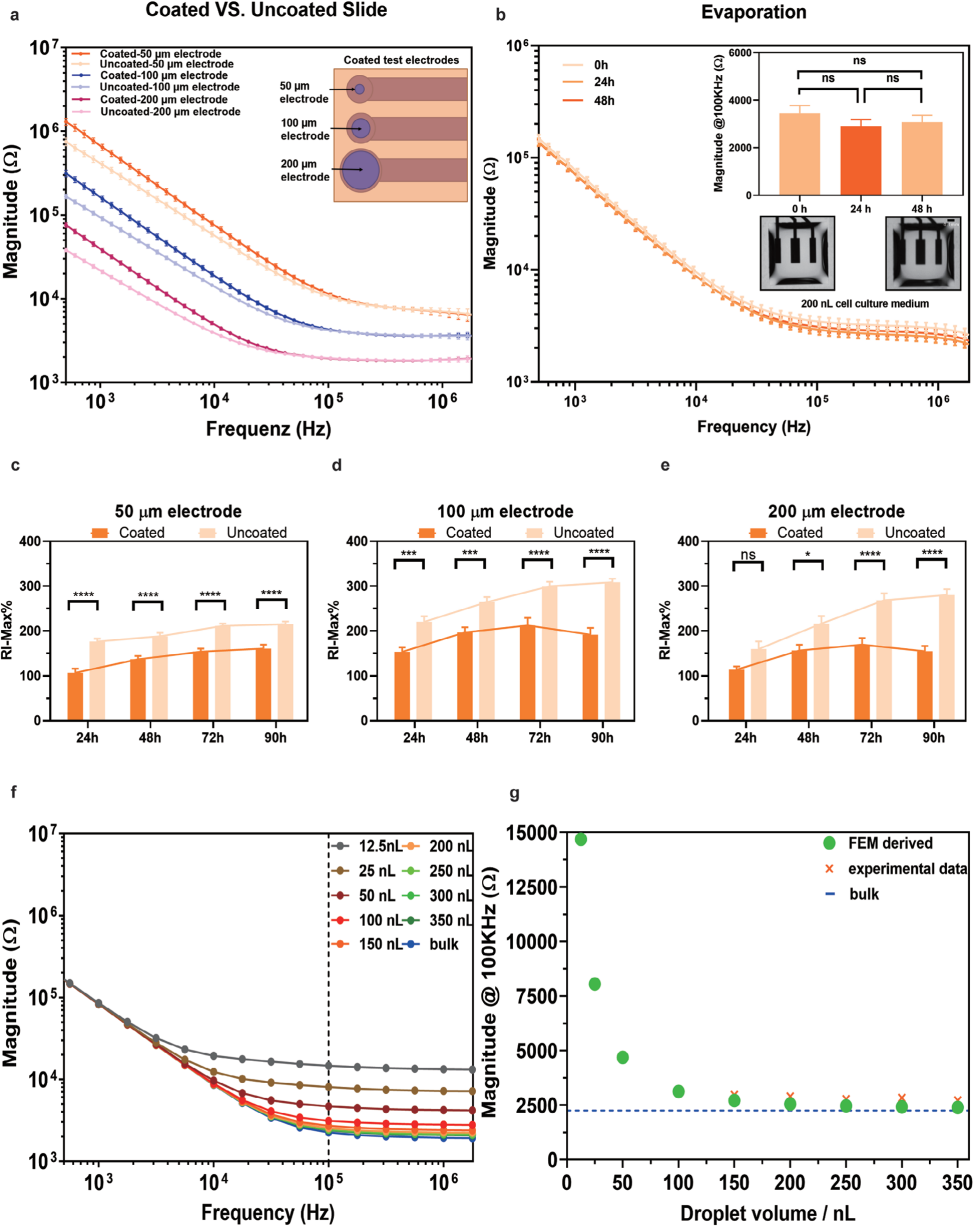
Impedance spectroscopy‐based characterization of eDMA. a) Comparison of the measurements of impedance conducted on round electrodes with diameters of 50 µm, 100 µm, and 200 µm, both uncoated and coated with a hydrophilic coating, within a 1 mL medium. The variation in impedance magnitude among electrodes, wherein impedance decreases with increasing electrode size, can be attributed to the larger surface area of the bigger electrodes. This increased surface area can accommodate a greater number of charge carriers, thereby reducing resistance to the flow of electrical current and resulting in decreased impedance (n = 15 electrodes). b) Impedance measurements obtained from 200 nL droplets of cell culture medium on eDMA at 0, 24, and 48 h, and corresponding microscope images of the droplets, demonstrating the possibility of continuous monitoring of stabile impedance signal on the eDMA platform for 48 h (n = 3 electrodes). c – e) RI‐Max% measurements of HEK293 cells cultured on coated and uncoated round electrodes with diameters of 50 µm, 100 µm, and 200 µm for 90 h (n = 12 electrodes) f) The impedance magnitude spectra derived from FEM simulation model corresponding to droplets of different volumes ranging from 12.5 to 350 nL. g) Comparison of impedance magnitudes obtained experimentally at 100 KHz in droplets of different volumes and in FEM simulation. (mean ± sem, *p ≤ 0.05, **p ≤ 0.01, ***p ≤ 0.001, ****p ≤ 0.0001).

Measuring impedance with eDMA differs from other state‐of‐the‐art platforms in two ways. First, it requires a specialized coating on the electrodes to create the hydrophilic‐superhydrophobic pattern. Second, unlike standard microelectrode array (MEA) platforms, which employ microliter to milliliter volumes significantly larger than the electrodes, eDMA utilizes nanoliter droplets that are comparable in size to the single electrode. As a consequence, the small size and shape of the droplets can potentially affect the impedance measurements. To ensure the reliability and accuracy of impedance measurements using the eDMA system, we investigated several parameters that could potentially influence measurement quality (**Figure** **2**; Figures S2 and S3, Supporting Information).

#### 2.2.1 Influence of Coating on Impedance Measurement

The influence of the hydrophilic nanoparticle‐based coating (*coated eDMA*) on impedance signal was investigated by measuring the impedance on coated and uncoated electrodes of different diameters, 50 µm, 100 µm, and 200 µm, in 1 mL of cell culture medium (Figure 2a; Figure S2, Supporting Information). The results revealed that at low frequencies, coated eDMA showed an increase in impedance magnitude compared to uncoated eDMA, while no significant difference was observed at higher frequencies (Figure 2a). An equivalent circuit‐based fitting of the impedance magnitude and phase angle spectra using simplified circuit model of the electrode‐electrolyte‐interface^[^ ^44^ ^]^ indicated that the hydrophilic coating primarily alters the properties of the electrodes towards a more ideal capacitance without significantly impacting their conductive capabilities (Figure S2, for more explanation see Supporting Information). This means that the integrity of impedance measurements of coated electrode is preserved, ensuring robust and reliable measurements.

Next, we examined whether a hydrophilic coating affects the monitoring of cell growth via impedance spectroscopy. Human Embryonic Kidney 293A cells (HEK293A) were seeded onto circular electrodes (50 µm, 100 µm, 200 µm) and cultured in a cell incubator for durations of 24, 48, 72, and 90 h, as shown in Figure 2c‐e. We then compared the relative impedance maximum (RI‐Max%) of cells cultured on coated versus uncoated electrodes. RI‐Max% is an impedance measured at a single frequency, at which given cells show maximum impedance signal, and since it is relative, normalized against a control, it reflects the difference between cells on coated versus uncoated electrodes. Our results revealed generally lower signals from the coated electrodes across all sizes and times. However, the growth trends observed by impedance spectroscopy were similar for both electrode types, suggesting that despite the slightly lower impedance signals from coated electrodes, the hydrophilic coating does not significantly interfere with the ability to monitor cell growth through impedance measurements. These results confirm the compatibility of the coating with impedance‐based cell growth monitoring.

#### 2.2.2 Influence of Droplet Curvature and Volume on Impedance Measurement

The standard droplet volume for cell culture application on the DMA platform is 200 nL confined on a 1 mm^2^ square area, the height of such droplet in the middle is ≈0.3 mm, which is comparable with the size of the electrodes. Such small liquid layer over the electrodes is not common in standard MEA measurements and can influence the impedance measurements of the electrodes. To investigate the influence of different volumes on impedance measurements, we first employed finite element method (FEM) simulations to model signal outputs from droplets of various volumes, aligning this theoretical framework with experimental data, as demonstrated in Figure 2f,g and Figure S3f,g (Supporting Information). The simulations reveal that as the droplet volume decreases from 300 to 25 nL, the potential difference across the droplet becomes more pronounced, especially near the electrodes, leading to a stronger potential gradient with decreasing volume. The current density is more concentrated at the electrode edges in smaller droplets, resulting in a significant increase at the electrode‐droplet interface and highlighting uneven current distribution. These findings suggest that smaller droplet volumes (less than 150 nL) intensify the focus of electric fields and current density at their interfaces, which leads to higher impedance, lower sensitivity and could compromise the accuracy of impedance measurements due to non‐uniform current distribution (Figure S3f, for more explanation see Supporting Information). The FEM simulation model was used to calculate impedance magnitude and phase angle spectra, which fitt well with experimentally measured impedance spectra (Figure S3g, Supporting Information). The simulation derived impedance magnitude spectra for droplets ranging from volumes of 12.5 nL to 350 nL showed no significant increase of impedance between volumes of 150 and 300 nL (Figure 2f). A comparison of experimental measurements and simulation data at 100 kHz using all three electrode pairs showed good concordance across all volumes (Figure 2g; Figure S3d, Supporting Information), confirming that measurement in droplets above 150 nL are reliable, and volume variations between 150 and 350 nL do not adversely affect the measurements. We also evaluated the consistency and reliability of the measured signal for repeated use of the same eDMA (Figure S3a,b, Supporting Information) and compared the signal differences between different eDMAs (Figure S3c, Supporting Information). Impedance measurements taken over five consecutive days and at 1‐minute intervals over 5 min on the same eDMA showed no significant variance in impedance values across different time points (Figure S3a,b, Supporting Information). Additionally, when comparing impedance measurements across various eDMAs, we observed slight differences at low frequencies, but no significant differences at high frequencies (Figure S3c, Supporting Information). Since cell density estimation relies on impedance values obtained at high frequencies, the impact of variations between different eDMAs is minimal. Moreover, by performing relative impedance measurements in cell studies and conducting blank measurements on each eDMA before each experiment, we normalize the cell measurement data to the corresponding blank impedance. This normalization ensures that any deviations between different eDMAs do not affect the final experimental results, highlighting the robustness of the eDMA platform.

For nanoliter droplet array, evaporation is a big challenge that can impact stability of impedance signal and wellbeing of the cells, particularly during real‐time monitoring over 24–48 hours. To minimize evaporation, we have designed a special humidity chamber for real‐time impedance measurements for up to 48 h (Figure S5, Supporting Information). We have then monitored the impedance in 200 nL of cell culture medium for 48 h. Figure 2b shows the results of the impedance and maximum impedance measurements, as well as images of droplets, after 0, 24, and 48 h, demonstrating no significant difference in obtained values, indicating the stability of the droplets, and therefore obtained impedance signal over 48 hours of measurements.

In summary, our demonstration confirms that the eDMA platform can reliably monitor impedance signals within volumes ranging from 150 to 350 nL for up to 48 h.

### 2.3 Measuring Impedance of Cells on eDMA

**Figure 3:**
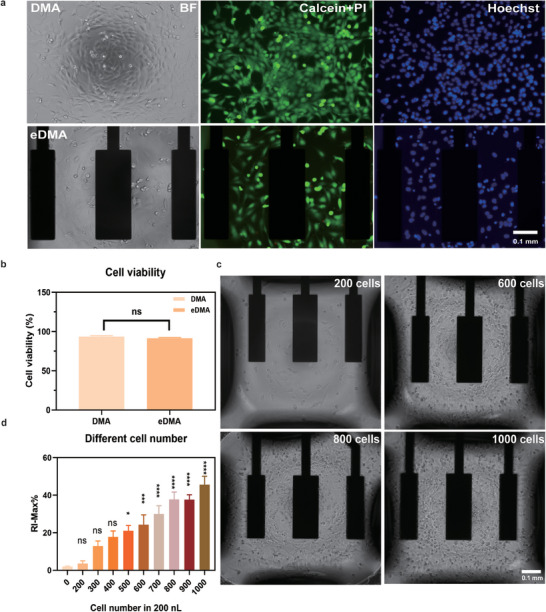
Measuring impedance of cells on eDMA. a) Microscope images of Hela CCL2 cells cultured on DMA and eDMA for 24 h (blue: Hoechst staining; green: Calcein AM staining; red: PI staining). b) Comparison of viability of Hela CCL2 cells cultured on DMA and eDMA for 24 hours (n = 3). c) Representative microscope images of different number of Hela CCL2 cells per droplet. d) Maximum relative impedance (RI‐Max%) measured in droplets containing different number of cells (n = 6 electrodes) (mean ± sem, *p ≤ 0.05, **p ≤ 0.01, ***p ≤ 0.001, ****p ≤ 0.0001).

Having demonstrated the feasibility of measuring the impedance in nanoliter droplets on eDMA,  we measured impedance of cells cultured in droplets formed on electrodes as the next step. First, we demonstrated the compatibility of the eDMA platform with cell culture in general. For this, we cultured adherent cervical carcinoma Hela CCL2 and suspension B cell lymphoma SU‐DHL4 cells on eDMA and standard DMA for 24 hours and compared morphology and viability of the cells on both platforms (**Figure** **3**; Figure S4, Supporting Information). Our results showed no difference in cell behavior between both platforms, with the viability of both cell types exceeding 95% (Figure 3; Figure S4, Supporting Information).

Impedance measurement is one of the effective techniques for quantifying cell numbers.^[^ ^46^, ^47^ ^]^ Further experiments were then conducted to explore the relationship between relative impedance and cell density on eDMA. First, Hela cells, ranging from 200 to 1000 cells per 200 nL droplet, were cultured until monolayer formation (24 hrs), followed by the impedance measurement. To compare the impedance obtained from droplets containing different numbers of cells we have extracted maximum relative impedance values RI‐Max% (Figure 3d). The maximum relative impedance showed a positive linear correlation between the number of cells and relative impedance (Figure 3c,d). For SU‐DHL4 cells, on the other hand, we found no significant correlation between the number of cells and relative impedance (Figure S4c,d, Supporting Information), as expected for cells of suspension nature that are not adhering on the electrodes and therefore could not be measured by using impedance spectroscopy also in conventional plate‐based systems in large volumes. Thus, we have demonstrated the feasibility of measuring the impedance signal from as low as 500 adherent cells in 200 nL droplets on eDMA.

### 2.4 Real‐Time Monitoring of Cell Culture on eDMA

**Figure 4:**
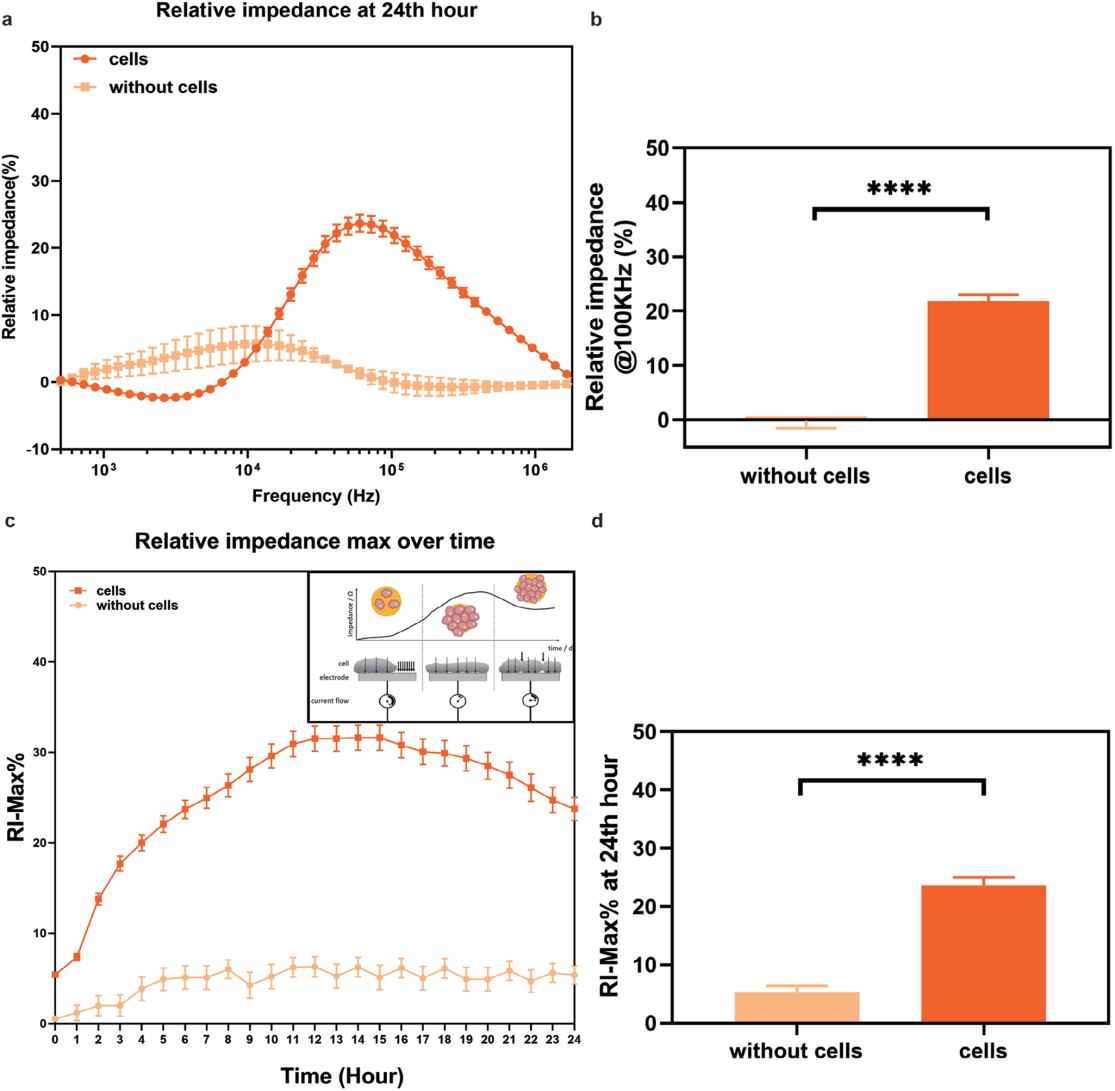
Real‐time monitoring of cell culture on eDMA. a,b) Impedance measurements of cells with maximum relative impedance (RI‐Max%) pick at ≈100 kHz in comparison with impedance obtained from droplets without cells. The measurement presented was extracted at the 24‐hour time point. c) Continuous monitoring of RI‐Max% of cells versus empty droplets over 24 h of culture d) Extracted values of RI‐Max% obtained from droplets with cells and cell culture medium at 24 h of culture. (n = 23 electrodes for cells, n = 7 electrodes for without cells, mean ± sem, *p ≤ 0.05, **p ≤ 0.01, ***p ≤ 0.001, ****p ≤ 0.0001).

As the next step, we monitored the culture of Hela CCL2 cells on eDMA for 24 h using EIS (**Figure** **4**). Our analysis revealed that in comparison with medium droplets without cells, the droplets with cells showed a significant increase in relative impedance at frequency of ≈100k Hz (Figure 4a,b). Figure 4c,d shows the maximum relative impedance (RI‐Max%) of cells in comparison with empty medium droplets over 24 hours of culture. We have observed a rapid increase of impedance in the first 4 h of culture, reflecting the initial settling and adhesion of cells onto the electrode's surface. Between 4 and 15 h, RI‐Max% was continuously increasing due to cell proliferation (Figure 4c, left panel), leading to a decrease in current flow and a corresponding increase in impedance. At the 15th hour, a nearly confluent cell layer had formed (Figure 4c, middle panel). Afterwards, Hela cells keep proliferating, which leads to decrease in cell sizes and increase in cell‐cell contacts on the same electrode area (Figure 4c, right panel). This leads to an increase in current flow and decrease in impedance in comparison to the state where fewer cells with higher cell surface area cover the same electrode area (Figure 4c, middle panel). This decrease in impedance after ≈15 h of incubation has been previously reported in the literature and may be influenced by cell size.^[^ ^48^ ^]^ Larger cells, such as HeLa cells, cover the surface area more quickly and reach confluence sooner than smaller cells, leading to an earlier phase of impedance decrease due to cell shrinkage.

The time required for impedance to reach a plateau is closely related to the initial number of cells. According to a previous study,^[^ ^49^ ^]^ different initial cell densities can lead to varying times for reaching the plateau phase during proliferation, depending on the cell type and initial density. Specifically, a higher initial seeding density results in an earlier appearance of the plateau phase, while a lower initial seeding density delays its onset. In our study, we observed that seeding 500 cells resulted in impedance values significantly different from those of a no‐cell control after 24 h (Figure 3d). This finding suggests that, at lower initial densities, cells require more time to induce significant changes in impedance. In summary, our results are in agreement with the previous study confirming that a higher initial seeding density accelerates the appearance of the plateau phase, in comparison to a lower initial seeding density.

To investigate the accuracy of our results, we performed weighted (1/σ2) linear fitting of only medium data (without cells) from 0 to 5 h and from 5 to 24 h to assess electrical drift (Figure S3e, Supporting Information). Electrical drift refers to a gradual change in baseline impedance readings over time, which can result from various factors such as temperature fluctuations, changes in the medium, and electrode degradation. This drift can obscure the true changes in impedance due to cell growth and behavior. From 0 to 5 h, the slope of the linear fit was 0.78 ± 0.08, indicating an initial increase in impedance (Figure S3e, Supporting Information). This suggests that some drift may be present during the initial stabilization phase. However, the impedance increase observed during the initial five hours of cell seeding on the electrodes was mainly attributed to the attachment of cells to the electrodes. As can be seen in Figure 4c, the impedance change caused by cells in the first 5 h, is significantly higher than the impedance observed in the absence of cells. Therefore, within the first five hours, the impedance increase due to the cell attachment can effectively overshadow an electrical drift that occurs without presence of the cells (Figure 4c). From 5 to 24 h, the slope was 0.008 percent per hour, which is negligible with regard to the measured maximum cell signal traces, indicating the system is stable at least for the experiment time ranges of 1 day that were used in this study (Figure S3e, Supporting Information).

As a result, we successfully demonstrated the continuous monitoring of cell culture on eDMA for a 24‐hour period utilizing impedance spectroscopy.

### 2.5 Real‐Time Monitoring the Response of Cells to Drugs on eDMA

**Figure 5:**
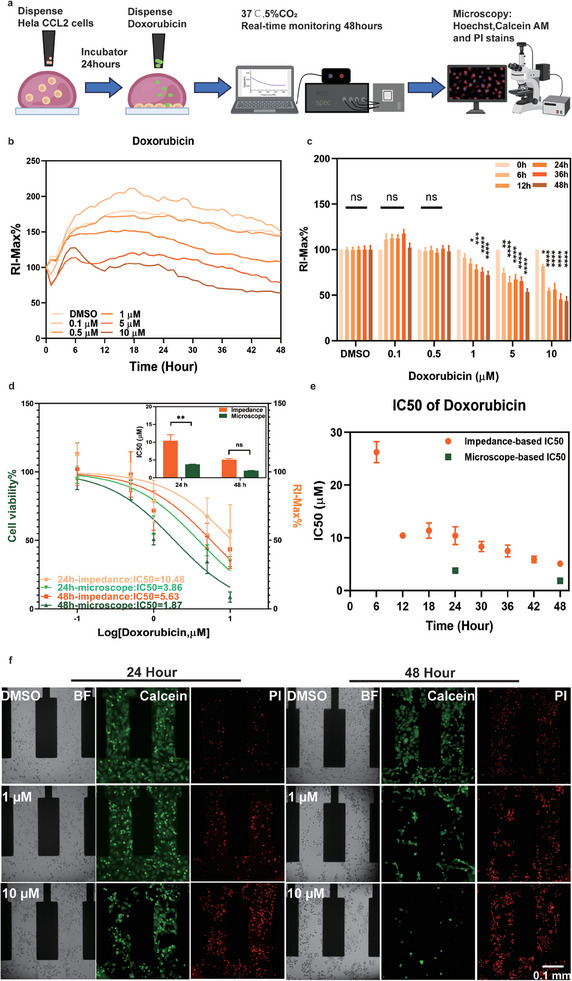
Real‐time monitoring drug response of cells on eDMA. a) Experimental workflow of real‐time monitoring drug response of cells on eDMA platform. b) Continuous RI‐Max% measurements from HeLa CLL2 cells treated with different concentrations of DOX over 48 h. c) Extracted values of RI‐Max% at 6 h, 12 h, 24 h, 36 h, and 48 h for different concentrations of DOX and vehicle control. Significance indicates the comparison of each time point within the concentration groups to time zero. d) Concentration‐dependent curves and calculated IC_50_ values using EIS and microscopy at 24 and 48 h of incubation. Impedance based IC_50_ value at 24 h was 10.48 µM with 95% confidence interval between 7.40 and 15.21 µM; and at 48 hours – 5.63 µM with 95% confidence interval between 4.12 and 7.72 µM. Microscope based IC50 value at 24 hours was 3.86 µM with 95% confidence interval between 2.79 and 5.31 µM; and at 48 h – 1.87 µM with 95% confidence interval between 1.23 and 2.85 µM. e) Estimated IC_50_ values at 6‐hour interval using EIS in comparison with IC50 values at 24 and 48 h using microscopy. f) Representative microscope images of HeLa CLL2 cells treated with different concentrations of DOX (Green: Calcein AM; Red: PI). DMSO: n = 10; 0.1 µM: n = 6; 0.5 µM and 5 µM: n = 7; 1 µM: n = 12; 10 µM: n = 9 electrodes. Microscopy: n = 3 (mean ± sem, *p ≤ 0.05, **p ≤ 0.01, ***p ≤ 0.001, ****p ≤ 0.0001).

To demonstrate the eDMA platform's capability for real‐time monitoring of drug response, we treated Hela CCL2 cells with an anticancer drug doxorubicin (DOX) on eDMA estimating drug response using impedance spectroscopy alongside microscopy (**Figure** **5**). The experimental workflow is presented in Figure 5a. Hela CCL2 cells, 600 cells per 200 nL droplet, were treated with a range of concentrations of DOX (0.1 µM, 0.5 µM, 1 µM, 5 µM, 10 µM) and impedance spectroscopy was recorded in real‐time over a 48‐hour period. Afterwards, the cell viability was assessed using live/dead staining with Calcein AM and Propidium Iodide (PI) followed by microscopy (Figure 5a). We observed a decreased in RI‐Max% as DOX concentrations increased (Figure 5b,c). During the initial 24 h of impedance monitoring in droplets with the vehicle control (DMSO), we observed a consistent increase in impedance, indicating cell growth. However, in the presence of DOX, cell growth was slower and exhibited a notable decline after 24 h, correlating with the drug concentration (Figure 5b,c; Table S1, Supporting Information). The end‐point analysis of cell viability after 24 and 48 h of treatment using microscopy revealed the concentration‐dependent decrease in cell viability as well (Figure 5f). We estimated and compared IC_50_ values obtained from impedance spectroscopy at different time points and microscopy after fixed 24 and 48 hours (Figure 5d,e). Our analysis revealed that IC_50_ of DOX using impedance spectroscopy was 10.48 µM (95% confidence interval (CI_95_) 7.40‐15.21 µM) and 5.63 µM (CI_95_ 4.12‐7.72 µM) at 24 and 48 h, respectively. In comparison, the IC_50_ derived from microscopy‐based readout were 3.86 µM (CI_95_ 2.79‐5.31 µM) at 24 h and 1.87 µM (CI_95_ 1.22‐2.85 µM) at 48 h. Overall, the determined IC_50_ values of both methods were in the same range, whereas microscopic‐determined IC_50_ values were in general lower with no significant difference for 48 h while significant for 24 h (Figure 5d inset). This suggests that the eDMA platform aligns more closely with traditional microscopy at later time point. This is due to the limitations of endpoint methods such as microscopy, which do not provide insights into intermediate points. We used a consistent approach to calculate IC50 values at 6‐hour intervals using maximum relative impedance data (Figure 5e), demonstrating the possibility to extract the information about the drug response at any time of experiment, which is not possible with endpoint assays as live/dead staining and microscopy (Figure 5e). In conclusion, our findings underscore the eDMA platform's efficiency in real‐time label‐free and non‐invasive monitoring of cell drug responses within a nanoliter format.

## 3 Conclusion

In the current study, we demonstrate a miniaturized platform for real‐time and label‐free monitoring of cell signal, which we have named eDMA. This system enables parallel measurements of impedance, reflecting cell growth within 200 nL droplets through the use of impedance spectroscopy. This study presents the first demonstration of continuous impedance monitoring within nanoliter droplets for up to 48 h. Using eDMA, we were able to accurately measure impedance deriving from as few as 500 cells cultured in 200 nL droplets, and successfully demonstrated parallel label‐free, real‐time monitoring of cellular drug response over a 48‐hour period. These capabilities were validated against traditional microscopy‐based assays, highlighting its ability to capture dynamic drug responses in real‐time and label‐free, even with low cell numbers within nanoliter droplets. This technology therefore offers significant potential for assessing the drug response of cells with limited availability, as for example physiologically relevant cells, such as patient‐derived primary cells or challenging‐to‐culture stem cells. Our system presents itself as a potent tool in drug discovery and personalized medicine, where real‐time data and miniaturized sample requirements are crucial. Further improvement of the platform, including enhanced electrode design and optimized hydrophilic‐superhydrophobic patterns, could improve both sensitivity and throughput. These enhancements would expand the platform's applications, making it a powerful tool for automation and high‐throughput screenings across a diverse spectrum of cell‐based assays. Overall, eDMA offers significant potential for advancing real‐time, label‐free analysis in drug discovery and personalized medicine.

## 4 Experimental Section

### Fabrication of eDMA (Electrode Droplet‐Microarray)

The printed electrode layer for eDMA was fabricated as it has been reported in previously published papers for fabrication of MEA slides.^[^ ^44^ ^]^ To clean the glass substrate surface (Borofloat 49/49/1.1 mm, Goettgens Industriearmaturen, Germany), we used ultrapure water, acetone, isopropanol, and ultrapure water, each for 2 min, in a pulsed ultrasonic bath. This was followed by a piranha etch and a final rinse with ultrapure water to ensure thorough cleaning. Next, the substrates were spin‐coated with a negative resist (AR‐N 4340, Allresist, Germany) to achieve a coating thickness of 2 µm. The photoresist was exposed for 8 s of UV light using a mask aligner (MA6, SÜSS MicroTec GmbH) and a photomask (Compugraphics Jena GmbH). After post‐baking on a hot plate at 100 °C for 2 min, the substrates were developed in AR 300–475 for 60 s, followed by a rinse with ultrapure water to stop the development process. Finally, a 50 nm indium tin oxide adhesion layer (EvoChem, Germany) was sputtered using a CREAMET 500 system (CREAVAC GmbH, Germany). To fabricate conductive layer, a 350 nm thick gold layer was deposited by sputtering at 350 W (DC) for 3 min, after which the photoresist layer was removed with acetone. Then, the gold‐coated substrates were heated on a hot plate at 200 °C for 60 min, then cooled to room temperature. Next, 1 mL of SU8‐2 (MicroChem Corp, United States) was spin‐coated onto each layer. The SU8‐2 was then heated to 65 °C for 1 min and then to 95 °C for 1 min. Exposure to UV define the desired pattern was performed using a photomask with an exposure time of 3.5 s. After exposure it was baked at 65 °C for 1 min and at 95 °C for 1 min. This was followed by development in mr‐DeV 600 for 40 s, which removed the unexposed SU‐8 layer covered with photomask in previous step, thereby revealing the electrode areas. The MEA was plasma treated (CREAMET 500, Germany) at 400 mA in an Ar atmosphere for 450 s and finally hard baked on a hotplate at 200 °C for 3 min. This resulted in a 1 µm SU8 layer.

Afterwards hydrophilic‐superhydrophobic pattern was created on the surface of MEA according to previously published work^[^ ^31^, ^45^ ^]^ by Aquarray GmbH. Briefly, first, MEA was activated by using ozone and coated with silica nanoparticle layer. The pattern of hydrophilic spots was created by using a photomask and fluorinating the coating to create superhydrophobic background. In this study we have used an array of 4 × 5 1 mm^2^ square hydrophilic spots, in total 20 hydrophilic spots, each covering 3 electrodes (Figure 1b–d).

### Electrochemical Characteristics Experiment

*Coated and Uncoated eDMA*: Hydrophilic coatings from Aquarray GmbH were applied to fabricate coated MEA chips. According to the standard protocol the uncoated eDMA was coated with Collagen I for enhanced cell growth. For each array 1 mL of medium was used to measure the impedance. HEK293A cells were cultured on top of MEA to study the effects of the hydrophilic coating on cell impedance signals. Impedance was continuously recorded at 24, 48, 72, and 90 h.

*Investigating Homogeneity of Impedance Signal in Different Volumes on eDMA*: 200 nL of PBS was dispensed onto each spot, and the impedance was recorded over a ten‐minute period. The impedance values from different electrodes at the ten‐minute mark were then compared. Additionally, varying volumes of PBS ranging from 150 to 350 nL were printed on each spot of the eDMA, and the impedance was recorded for 10 min. The impedance values at the ten‐minute mark for these different volumes were also compared to assess any variations.

*Reusability of eDMA*: The impedance measurements were performed in 200 nL of PBS five times in one day and 200 nL and once daily across five separate days. Each followed by a ten‐minute impedance measurement. Impedance values recorded at the ten‐minute mark for each eDMA were compared to evaluate the consistency and durability of the eDMA over multiple uses both within a single day and over an extended period.

### Cell Culture and Drug Treatment on eDMA

Hela CCL2 cells and SU‐DHL4 cells were used for the study. The Hela CCL2 cells were cultured in DMEM medium supplemented with 10% FBS and 1% Penicillin‐Streptomycin on 10 cm^2^ Petri dish. The SU‐DHL4 cells were cultured in RPMI medium supplemented with 10% FBS and1% Penicillin‐Streptomycin on 10 cm^2^ Petri dish. Both cell lines were split once every 2–3 days.

eDMA was sterilized with 100% ethanol for 2 min and dried under clean bench. For cell culture experiment, HeLa CCL2 cells in density of 3 × 10^6^ per mL were dispensed on eDMA by using non‐contact dispenser I‐DOT One (Dispendix GmbH, Stuttgart, Germany). Afterwards the humidity chamber was attached onto eDMA to build a humidified environment (Figure S5, Supporting Information). The humidity chamber was equipped with humidity pads and PBS to avoid evaporation of droplets during culture. Then eDMA were placed into impedance spectroscope to measure impedance for 24 h. For drug treatment experiment, cells were seeded as described previously.^[^ ^50^ ^]^ After 24 h, cells were respectively treated by 0.1, 0.5, 1, 5, 10 µM of doxorubicin. Then eDMA with humidity chamber attached was placed into impedance spectroscope to measure impedance for 48 h.

### Impedance Spectroscopy

Impedance of the cells was measured and calculated as described previously.^[^ ^51^ ^]^ The measurement system consists of a self‐developed multiplexer system and the high precision impedance analyzer ISX‐3 (Sciospec Scientific Instruments, Germany).

### Cell Viability Assay

After the cell impedance measurement, cells were stained by using 5 µg mL^−1^ Hoechst 33342, Calcein AM, and propidium iodide (PI) for 15 min at 37 °C. Cells were imaged with Leica Thunder 3D Imager microscope (Leica, Hillsboro, USA) and Keyence VHX‐7000 microscope (KEYENCE, Osaka, Japan).

### Statistical Analysis

The experimental data in this research were based on at least 3 independent experiments. All Impedance spectroscope data were analyzed by using software IDAT v4. The relative impedance was calculated as follows: (Z_with cells –_ Z_blank_) / Z_blank_ × 100%. The peak values (maximum amplitude) of the relative impedance, denoted as RI‐Max%, percentage were then continuously monitored over time. All experimental data were analyzed and compared using the t‐test and one‐way ANOVA. All statistical tests in this research were performed using GraphPad Prism 8 (San Diego, CA).

## Conflict of Interest

The authors declare no conflict of interest.

## Supporting information



Supporting Information

## Data Availability

The data that support the findings of this study are available under DOI https://doi.org/10.35097/wg6pznvxs279q7g3.
