# Supplementary material for: Electrode Droplet Microarray (eDMA): An Impedance Platform for Label‐Free Parallel Monitoring of Cellular Drug Response in Nanoliter Droplets
Source: Adv Healthc Mater. 2024 Oct 15;14(3):2402046. doi: 10.1002/adhm.202402046 (PMC11773094; doi:10.1002/adhm.202402046)
Supplement: Supplementary file 1 — Supporting Information [file ADHM-14-0-s001.docx]

Supporting Information

Electrode Droplet Microarray (eDMA): an impedance platform for label-free parallel monitoring of cellular drug response in nanoliter droplets

Meijun Zhou, Joaquin E. Urrutia Gomez, Nikolaj K. Mandsberg, Sida Liu, Sabine Schmidt, Matthias Meier,Pavel A. Levkin*, Heinz-Georg Jahnke* and Anna Popova*

E-mail: [anna.popova@kit.edu](mailto:anna.popova@kit.edu); [heinz-georg.jahnke@bbz.uni-leipzig.de](mailto:heinz-georg.jahnke@bbz.uni-leipzig.de); [levkin@kit.edu](mailto:levkin@kit.edu)

**Table of Contents**

[Figure S1. The design and impedance measurements on eDMA. 4](#_Toc174608002)

[Figure S2. The equivalent circuit model of coated and uncoated electrodes. 5](#_Toc174608003)

[Figure S3. Electrochemical characteristics of eDMA. 7](#_Toc174608004)

[Figure S4. Measuring impedance of suspension cells on eDMA.. 9](#_Toc174608005)

[Figure S5. The design of humidity chamber. 10](#_Toc174608006)

[Table S1. Statistical analysis for relative impedance of Doxorubicin-cell response 11](#_Toc174608007)

[Table S2. Statistical analysis for impedance of 5 days (Figure S3a) 13](#_Toc174608008)

[Table S3. Statistical analysis for impedance of 3 eDMAs (Figure S3b) 17](#_Toc174608009)

[Table S4. Statistical analysis for impedance of 3 eDMAs (Figure S3c) 21](#_Toc174608010)

**Experimental methods**

**Materials and Reagents**

60-electrodes microelectrode arrays (60-MEA) were manufactured by the Centre for Biotechnology and Biomedicine of Leipzig University. Dulbecco’s modified Eagle medium (DMEM), fetal bovine serum (FBS), penicillin–streptomycin, 0.25% trypsin/EDTA, Collagen Ⅰ, Hoechst33342, Calcein-AM and propidium iodide (PI) were acquired from Thermo Fisher Scientific. The Hela-CCL2 and SU-DHL4 cells were purchased from DSMZ GmbH and cultured in a way introduced by the company’s protocols. Doxorubicin was purchased from Sigma-Aldrich.

**Water contact angle measurement**

The water contact angles (WCAs) were measured as described previously.^[1]^ Typically, different volume of deionized water was placed on different droplet on eDMA, and WCAs were acquired by a Drop Shape Analyzer machine according to manufacture protocol (Krüss, Hamburg, Germany).

**Design and fabrication of humidity chamber**

Rhino software was utilized to create a 3D model of the humidity chamber, ensuring it meets the required specifications and functionality. Then, the Ultimaker 3 DLP printer was used for printing humidity chamber by using PLA link material.

**Equivalent circuit and finite element method (FEM) simulation model**

For equivalent circuit modelling, we have fit the measured impedance magnitude and phase angle spectrum. Then we used self-developed software based on the LabView development suite (National Instruments). The fitting of these spectra was proficiently executed through a Levenberg-Marquardt based constrained optimization algorithm.

For FEM stimulation model, the Finite Element Method (FEM) simulation, conducted using COMSOL Multiphysics 5.3 (Comsol Multiphysics GmbH) with its AC/DC module, used CAD 2016 to design droplets shape.


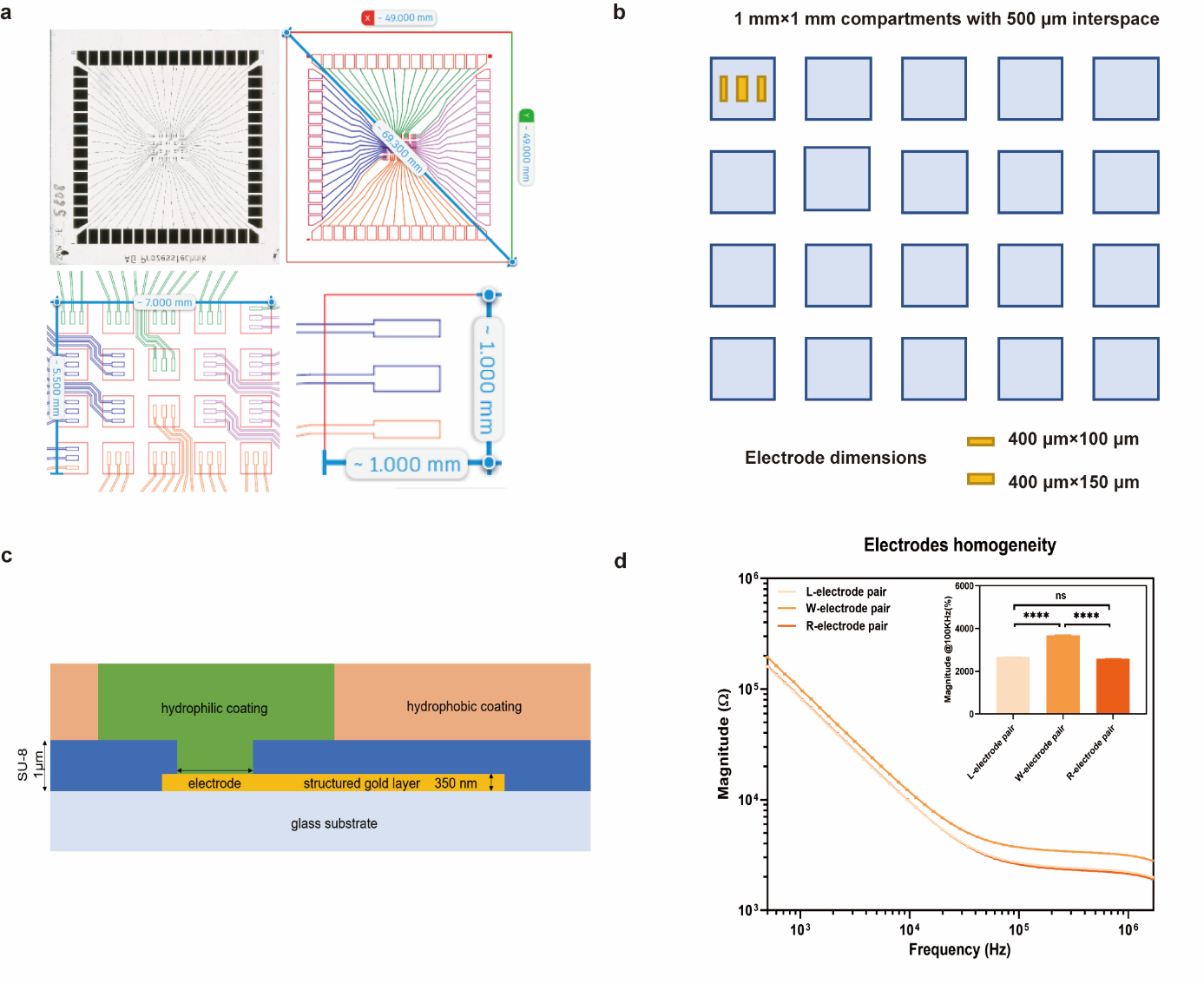


# Figure S1. The design and impedance measurements on eDMA. (a) The dimension of eDMA chip and hydrophilic-superhydrophobic array. As each DMA was made manually, each eDMA was assigned a unique serial number. (b) Each eDMA is composed of 20 spots, each measuring 1mm by 1mm. On every spot, there are three electrodes, which come in two different sizes. (c) The structure diagram of eDMA. (d) Analysis of homogeneity of impedance signal obtained from different electrodes. Comparing the variability of impedance signal obtained from different electrode pairs: L-electrode pair, R-electrode pair, and W-electrode pair (n=20 electrodes, mean ± sem, * P ≤ 0.05, ** P ≤ 0.01, *** P ≤ 0.001, **** P ≤ 0.0001). The impedance signal, measured in 200 nL droplets, which is standard volume used for cells culture, was stable.^[31]^ As expected, the impedance values obtained from the W electrode pair were significantly higher than those recorded from the L and R electrodes pairs, 3686.9 Ω vs 2624.1 Ω, which is attributed to the fact that the electrode area of the middle electrode is larger and distance over which current passes through the pairs involving the W electrode is longer than that of the L and R.


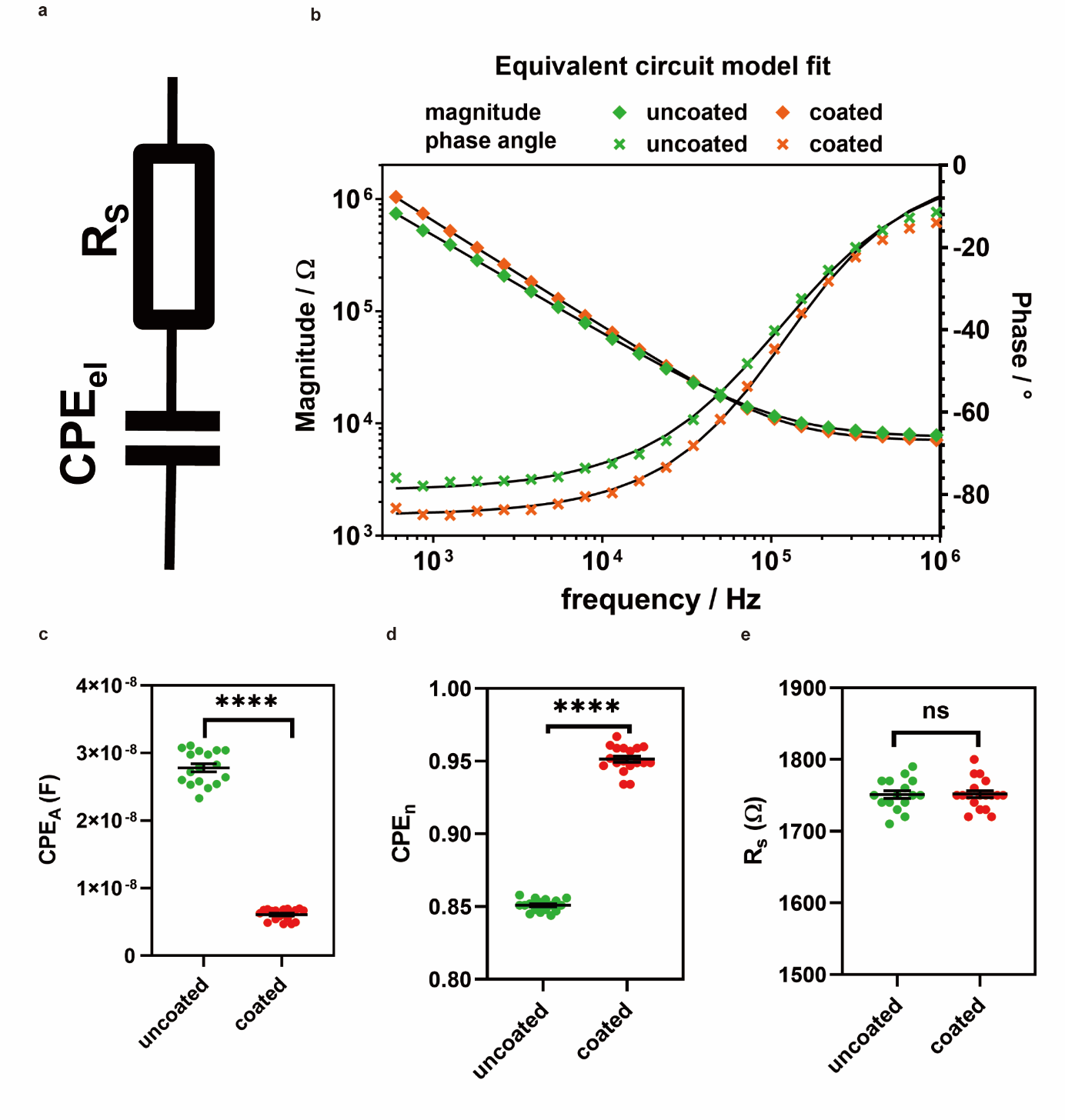


Figure S2. The equivalent circuit model of coated and uncoated electrodes. (a) Equivalent circuit model (ECM) for electrode-electrolyte interface. (b) Comparison of measured impedance magnitude and phase angle spectra (symbols) and ECM-fitting derived spectra (black lines). (c - e) Analysis of ECM parameter for capacitance (CPE_A_, n) and resistance (R_S_) (n=18, mean ± sem, * P ≤ 0.05, ** P ≤ 0.01, *** P ≤ 0.001, **** P ≤ 0.0001). The equivalent circuit model (ECM) consists of a constant phase element (CPE_el_) that reflects the non-ideal capacitive characteristics, in series with the spreading resistance (R_S_) observed across the electrode-electrolyte interface (**Figure S2a**). ECM derived impedance spectra fitting fits quite well to the measured impedance magnitude and phase angle of electrodes without cells (**Figure S2b**). Based on the ECM fittings, we statistically analyzed the values of CPE_A_, CPE_n_ and R_s_ (**Figure S2c-e**). The constant phase element CPE_el_ describes the non-ideal capacitance of the electrical double layer at the electrode-electrolyte interface with the parameter CPE_A_ (capacitance) and CPE_n_ that describes how closely the CPE behavior approximates an ideal capacitor. Such capacitors do not exist in real life. Values of CPE_n_ close to 1 indicate nearly ideal capacitive behavior of electrode, while values less than 1 indicate increasing deviation from ideal behavior due to e.g. surface roughness or inconsistencies. Our results show that coated electrodes have significantly lower CPE_A,_ 2.782*10^-8^ F vs. 6.093*10^-9^ F, but higher CPE_n,_ 0.8509 vs. 0.9514. Lower CPE_A_ indicates that the coated electrode reduces its effective capacitance, which could mean that the coating modifies the electrode's surface properties. Higher CPE_n_ however demonstrates that the hydrophilic coating results in electrodes being closer to ideal capacitance in comparison with uncoated electrodes (**Figure S2c, d).** All together this indicates that the coating leads to a more uniform and less obstructive surface, allowing for a more ideal capacitive behavior. R_s_ represents the real impedance encountered as electrical current moves from the boundaries of the electrode surface through the electrolyte. The obtained values of R_s_ from coated versus uncoated electrodes revealed no significant differences (**Figure S2e**). Thus our analysis suggests that while the hydrophilic coating modifies the capacitive properties of the electrodes, however without substantially affecting their ability to conduct current, hence maintaining the integrity of impedance measurements.


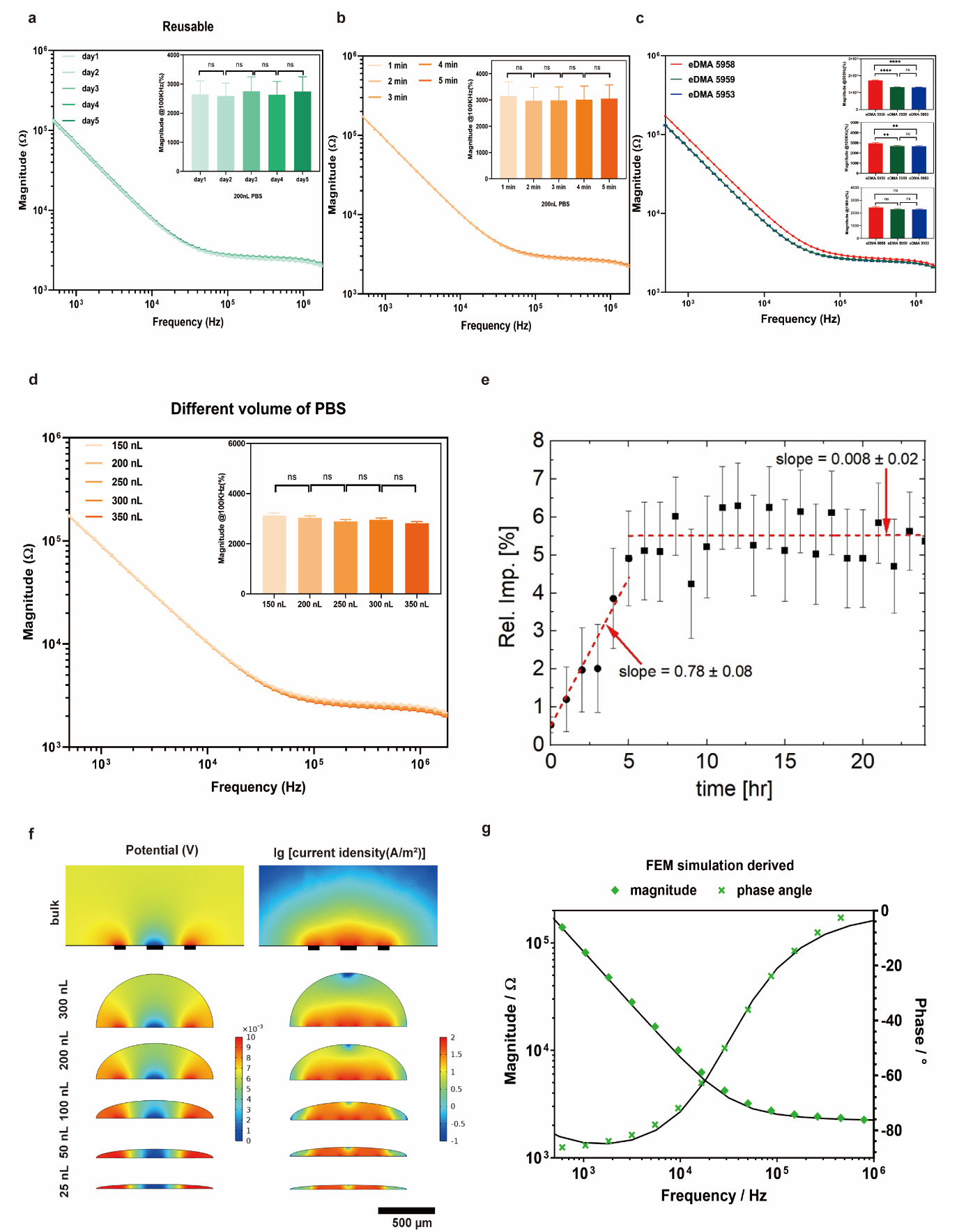


Figure S3. Electrochemical characteristics of eDMA. (a) Investigation of the reusability of eDMA. The impedance measurements were conducted in 200 nL of PBS over several days to evaluate the consistent performance of eDMA over time. The bar graph on the right shows impedance measurements at 100 KHz over several days (n=46 electrodes). (b) The average impedance measurements values across various frequencies obtained from 20 hydrophilic spots of control group (only PBS) for each minute over 5 minutes measurement. The bar graph on the right shows impedance measurements at 100 KHz over several minutes (n=58 electrodes). (c) The average impedance values across various frequencies obtained from 20 hydrophilic spots at eDMA 5959, eDMA 5958 and eDMA 5953. The bar graph on the right shows the impedance measurement of different eDMA at 500HZ, 100 KHz and 1MHz frequencies respectively (n=58 electrodes for eDMA 5958; n=55 electrodes for eDMA 5959; n=57 for eDMA 5953). (d) Examining variations in the impedance magnitude in different droplet volumes on eDMA (n=39 electrodes). (e) Electrical drift on eDMA for 24 hours (n=7 electrodes). (f) FEM simulation model derived potential and current density for different droplet volumes. (g) Comparison of FEM simulation derived impedance magnitude and phase angle spectra (black lines) and experimentally measured spectra (symbols) (mean ± sem, * P ≤ 0.05, ** P ≤ 0.01, *** P ≤ 0.001, **** P ≤ 0.0001). FEM allowed us to anticipate the behavior of different volume droplets, providing a theoretical framework to compare against experimental data. The calculated electric potentials demonstrate that as droplet volume decreases (from 300 nL to 25 nL), the potential difference notably increases, with the potential increasing at the edges near the electrodes and decreasing in the middle. This gradient in potential difference is more pronounced in smaller droplets due to the edges being in closer proximity to the electrodes (**Figure S3f**). These results indicate that, with decrease droplet volumes, the distribution of the electric field within the droplet becomes increasingly inhomogeneous, concentrating the field strength at the periphery while weakening it at the center. This uneven electric field distribution can negatively affect impedance measurements. The simulation results show that the current is slightly concentrated at the edge of the electrode. As the droplet volume decreases, the current density at the electrode-droplet interface increases significantly, the current density at the edge electrode increases significantly, which means current is unevenly distributed on the electrodes in smaller droplets (**Figure S3f**). All together, these suggest that smaller droplet volumes focus electric fields and current density at their interfaces, potentially affecting impedance measurements due to the non-uniform distribution of current.


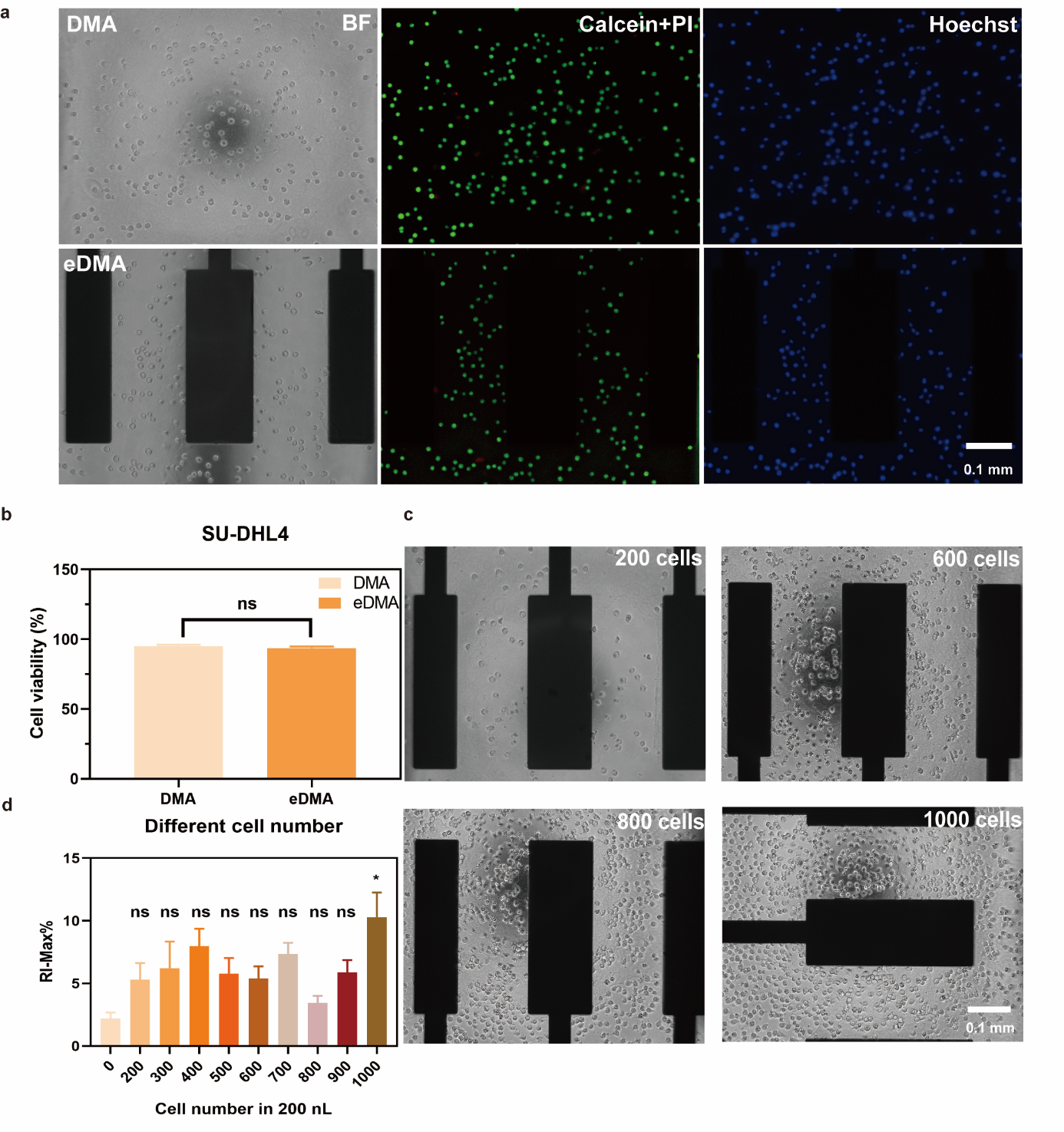


# Figure S4. Measuring impedance of suspension cells on eDMA. (a) Microscope images of SU-DHL4 cells cultured on DMA and eDMA for 24 hours (blue: Hoechst staining; green: Calcein AM staining; red: PI staining). (b) Comparison of viability of SU-DHL4 cells cultured on DMA and eDMA for 24 hours (n=3). (c) Representative microscope images of different number of SU-DHL4 cells per droplet. (d) Maximum relative impedance (RI-Max%) measured in droplets containing different number of SU-DHL-4 cells (n=6 electrodes) (mean ± sem, * P ≤ 0.05, ** P ≤ 0.01, *** P ≤ 0.001, **** P ≤ 0.0001).


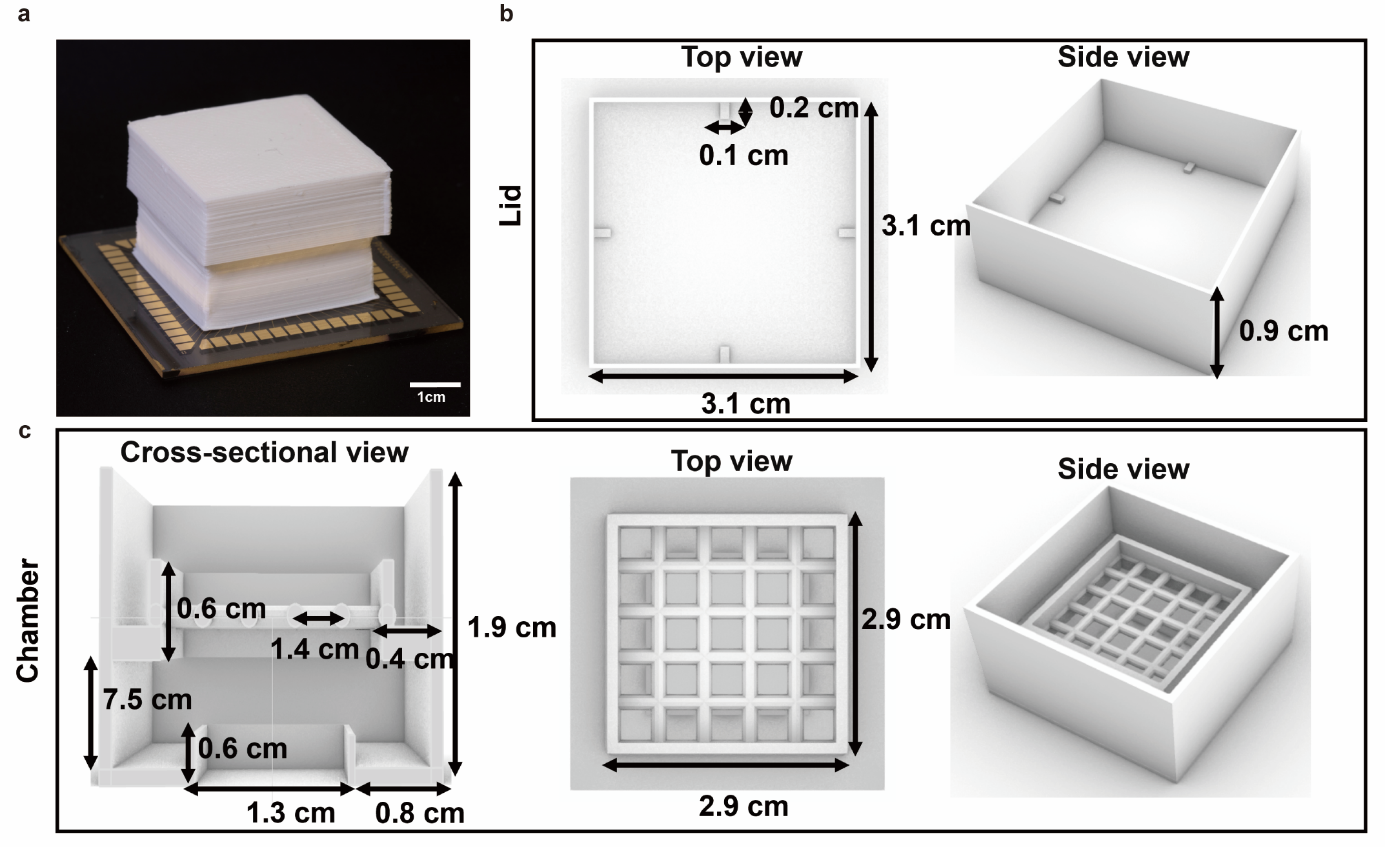


# Figure S5. The design of humidity chamber. (a) A photo of humidity chamber attached to eDMA. (b) The design of humidity chamber lid. Tissue pad was placed inside the lid and wetted with 1 mL PBS. (c) The design of humidity chamber. 2 mL of PBS was filled in all channels in the chamber.

Table S1. Statistical analysis for relative impedance of Doxorubicin-cell response **(Figure 5c)** (DMSO: n=10; 0.1 µM: n=6; 0.5 µM and 5 µM: n=7,1 µM: n=12,10 µM: n=9 electrodes).

| Hela CCL2 | RI-Max% (mean ± sem) | | | | | |
| --- | --- | --- | --- | --- | --- | --- |
| Hours | DMSO | 0.1  [µM] | 0.5  [µM] | 1  [µM] | 5  [µM] | 10  [µM] |
| 1 | 116.3±5.9 | 203.6±3.6 | 110.8±3.2 | 110.9±4.6 | 75.5±7.1 | 74.6±11.3 |
| 2 | 113.9±4.3 | 205.4±4.1 | 108.3±2.3 | 110.2±3.9 | 85.3±1.7 | 84.8±5.1 |
| 3 | 131.4±3.8 | 204.4±5.5 | 123.6±3.7 | 121.7±4.5 | 96.5±2.6 | 101.6±3.7 |
| 4 | 145.6±4 | 211.3±7.9 | 138.1±5.0 | 137.2±5.8 | 108.1±5.3 | 118.8±3.3 |
| 5 | 152.1±3.8 | 211.4±8.7 | 147.7±5.6 | 144.1±6.1 | 112.8±6.8 | 127.2±3.1 |
| 6 | 155.8±3.6 | 205.6±9.4 | 153.4±5.8 | 145.5±6.0 | 114.0±6.9 | 127.5±3.1 |
| 7 | 157.0±3.4 | 200.5±8.5 | 156.6±6.7 | 147.5±6.5 | 108.6±7.6 | 120.1±3.7 |
| 8 | 161.5±3.5 | 200.4±7.6 | 157.5±5.4 | 146.8±7.2 | 106.2±8.2 | 111.8±3.0 |
| 9 | 162.1±3.9 | 196.4±10.1 | 160.6±4.3 | 147.5±7.6 | 105.4±9.2 | 106.0±3.5 |
| 10 | 162.6±4.5 | 193.1±7.3 | 161.8±5.4 | 147.2±7.6 | 105.1±9.3 | 101.0±3.8 |
| 11 | 168.3±3.8 | 199.3±7.1 | 168.2±5.8 | 148.8±8.0 | 106.1±10.4 | 97.5±3.6 |
| 12 | 172.8±4.3 | 195.2±6.4 | 172.2±6.6 | 150.1±8.7 | 108.7±10.2 | 94.7±5.1 |
| 13 | 175.3±4.9 | 193.6±7.6 | 171.0±6.4 | 151.6±9.0 | 112.2±9.9 | 101.1±6.8 |
| 14 | 177.4±4.7 | 193.4±5.9 | 171.0±6.2 | 150.3±8.9 | 112.2±9.6 | 99.4±6.8 |
| 15 | 178.5±5.3 | 197.4±6.5 | 171.6±4.6 | 150.2±10.0 | 115.2±9.0 | 100.6±7.9 |
| 16 | 179.3±4.9 | 194.8±4.7 | 171.9±5.1 | 152.5±10.4 | 116.6±8.8 | 104.2±9.8 |
| 17 | 179.1±4.7 | 185.0±5.8 | 172.2±4.8 | 151.3±10.0 | 119.8±9.7 | 105.8±10.7 |
| 18 | 179.7±4.5 | 183.9±4.6 | 172.8±4.1 | 151.3±9.6 | 120.2±9.7 | 105.0±11.1 |
| 19 | 175.7±6.0 | 182.2±5.5 | 169.4±3.4 | 150.3±9.1 | 117.3±9.4 | 105.2±11.3 |
| 20 | 177.8±5.7 | 180.8±5.0 | 168.3±4.6 | 149.7±9.2 | 121.0±9.3 | 102.9±10.6 |
| 21 | 176.1±5.5 | 182.1±5.3 | 168.9±5.1 | 149.1±10.4 | 117.3±9.5 | 102.3±11.4 |
| 22 | 174.5±5.4 | 179.7±5.2 | 171.0±6.8 | 144.4±9.4 | 116.3±8.9 | 102.6±11.4 |
| 23 | 174.7±5.6 | 182.4±4.7 | 171.7±7.5 | 142.6±8.0 | 118.2±8.2 | 104.2±12.8 |
| 24 | 177.2±5.2 | 178.2±6.5 | 171.3±7.1 | 142.6±7.7 | 117.1±8.2 | 99.7±11.7 |
| 25 | 173.7±6.1 | 172.3±7.4 | 173.3±7.7 | 140.1±7.9 | 114.1±6.8 | 95.4±11.4 |
| 26 | 171.3±6.3 | 174.4±7.6 | 170.4±6.3 | 139.3±8.2 | 112.3±6.3 | 96.8±12.7 |
| 27 | 166.2±6.2 | 173.0±7.6 | 166.3±6.1 | 135.1±7.5 | 111.0±7.2 | 86.9±10.7 |
| 28 | 168.1±7.0 | 169.3±8.5 | 164.9±6.7 | 133.6±6.7 | 112.8±7.4 | 86.9±11.1 |
| 29 | 168.1±6.1 | 170.5±9.1 | 167.6±7.0 | 131.2±7.2 | 111.8±7.7 | 85.4±11.5 |
| 30 | 166.6±6.2 | 166.9±8.8 | 166.4±6.9 | 128.3±6.8 | 110.5±7.8 | 82.1±11.3 |
| 31 | 164.3±6.5 | 165.6±7.8 | 164.5±5.9 | 126.6±7.5 | 110.4±7.9 | 81.1±11.6 |
| 32 | 162.7±6.4 | 160.2±7.7 | 161.7±4.7 | 125.7±7.4 | 105.7±6.4 | 76.8±11.2 |
| 33 | 163.2±6.5 | 160.1±7.9 | 162.9±4.9 | 120.6±7.6 | 103.7±6.3 | 74.1±10.5 |
| 34 | 158.1±6.1 | 156.3±6.8 | 162.4±4.7 | 119.1±7.1 | 102.3±5.5 | 74.2±10.7 |
| 35 | 155.0±6.7 | 149.8±5.6 | 159.9±4.2 | 118.6±6.7 | 100.0±5.6 | 70.8±9.9 |
| 36 | 154.7±6.1 | 203.6±6.2 | 156.6±5.1 | 118.8±7.2 | 101.3±5.6 | 70.2±9.8 |
| 37 | 152.9±5.6 | 205.4±6.6 | 152.9±5.0 | 118.7±7.0 | 98.8±4.9 | 69.5±9.5 |
| 38 | 150.9±5.2 | 204.4±4.0 | 151.5±5.3 | 118.7±6.4 | 98.9±3.7 | 69.3±9.3 |
| 39 | 149.5±5.4 | 211.3±4.1 | 149.7±5.6 | 116.9±6.1 | 95.1±3.1 | 68.9±9.0 |
| 40 | 149.2±5.4 | 211.4±2.7 | 149.7±6.3 | 114.1±5.7 | 92.5±2.8 | 68.5±8.5 |
| 41 | 152.3±6.6 | 205.6±2.9 | 150.8±6.4 | 113.4±5.4 | 91.5±3.0 | 65.3±7.8 |
| 42 | 152.1±6.4 | 200.5±5.1 | 147.5±6.4 | 111.4±4.7 | 88.7±3.7 | 66.9±8.3 |
| 43 | 151.3±5.8 | 200.4±6.4 | 149.0±6.2 | 110.9±4.8 | 89.7±3.5 | 65.7±7.7 |
| 44 | 153.1±6.2 | 196.4±5.2 | 149.3±7.5 | 109.6±4.8 | 86.2±3.2 | 66.2±7.8 |
| 45 | 150.8±5.5 | 193.1±5.0 | 147.3±7.9 | 108.1±5.0 | 85.9±3.2 | 66.5±7.7 |
| 46 | 151.5±6.4 | 199.3±3.7 | 146.1±7.9 | 108.0±5.3 | 79.7±2.9 | 66.4±7.6 |
| 47 | 145.9±5.9 | 195.2±6.0 | 144.2±8.1 | 109.0±5.7 | 79.9±3.0 | 64.7±6.8 |
| 48 | 146.7±6.6 | 193.6±7.1 | 144.0±9.2 | 107.9±6.0 | 78.3±4.3 | 63.8±6.8 |

Table S2. Statistical analysis for impedance of 5 days (Figure S3a)

|  | day1 | | day2 | | day3 | | day4 | | day5 | |
| --- | --- | --- | --- | --- | --- | --- | --- | --- | --- | --- |
| Frequency  (Hz) | Mean  ±sem | N | Mean  ±sem | N | Mean  ±sem | N | Mean  ±sem | N | Mean  ±sem | N |
| 500 | 131,474.6  ±1,733.5 | 55 | 134,882.9  ±1,743.1 | 53 | 127,178.3  ±1,638.6 | 55 | 132,360.8  ±1,747.4 | 52 | 137,772.9  ±1,907.0 | 46 |
| 601 | 110,118.3  ±1,433.2 | 55 | 113,048.3  ±1,466.3 | 53 | 106,732.5  ±1,375.6 | 55 | 110,943.5  ±1,458.7 | 52 | 115,455.4  ±1,612.2 | 46 |
| 723 | 91,932.9  ±1,202.9 | 55 | 94,329.1  ±1,220.0 | 53 | 89,049.2  ±1,151.2 | 55 | 92,662.4  ±1,227.7 | 52 | 96,295.6  ±1,330.3 | 46 |
| 869 | 78,331.1  ±1,031.3 | 55 | 80,323.4  ±1,045.5 | 53 | 75,843.8  ±987.3 | 55 | 78,822.7  ±1,049.6 | 52 | 81,918.6  ±1,137.0 | 46 |
| 1045 | 64,373.8  ±839.2 | 55 | 66,055.6  ±853.6 | 53 | 62,354.0  ±801.4 | 55 | 64,811.6  ±854.4 | 52 | 67,372.4  ±931.9 | 46 |
| 1256 | 54,479.8  ±713.8 | 55 | 55,862.3  ±722.5 | 53 | 52,702.0  ±682.0 | 55 | 54,788.9  ±726.0 | 52 | 56,932.1  ±782.8 | 46 |
| 1510 | 45,250.4  ±590.4 | 55 | 46,401.4  ±600.0 | 53 | 43,789.8  ±565.9 | 55 | 45,510.8  ±602.6 | 52 | 47,292.3  ±653.4 | 46 |
| 1815 | 37,881.7  ±495.8 | 55 | 38,863.0  ±504.8 | 53 | 36,686.8  ±474.2 | 55 | 38,099.3  ±505.7 | 52 | 39,579.7  ±546.8 | 46 |
| 2183 | 31,743.4  ±416.7 | 55 | 32,559.9  ±423.0 | 53 | 30,746.8  ±399.4 | 55 | 31,923.6  ±423.6 | 52 | 33,167.8  ±459.0 | 46 |
| 2624 | 26,612.3  ±350.0 | 55 | 27,298.1  ±356.0 | 53 | 25,792.1  ±335.4 | 55 | 26,762.4  ±356.5 | 52 | 27,798.4  ±386.1 | 46 |
| 3155 | 22,307.0  ±294.4 | 55 | 22,881.7  ±299.5 | 53 | 21,639.8  ±283.7 | 55 | 22,432.7  ±299.6 | 52 | 23,298.1  ±324.7 | 46 |
| 3793 | 18,738.8  ±248.9 | 55 | 19,215.7  ±253.1 | 53 | 18,195.5  ±239.8 | 55 | 18,842.5  ±253.4 | 52 | 19,569.9  ±274.8 | 46 |
| 4560 | 15,741.5  ±210.9 | 55 | 16,137.6  ±214.1 | 53 | 15,303.1  ±203.7 | 55 | 15,830.5  ±214.5 | 52 | 16,435.6  ±232.8 | 46 |
| 5482 | 13,261.1  ±179.8 | 55 | 13,587.5  ±182.1 | 53 | 12,910.5  ±174.2 | 55 | 13,333.7  ±182.5 | 52 | 13,842.1  ±198.6 | 46 |
| 6591 | 11,194.2  ±154.0 | 55 | 11,461.4  ±155.7 | 53 | 10,921.4  ±150.1 | 55 | 11,255.6  ±156.3 | 52 | 11,682.5  ±170.3 | 46 |
| 7924 | 9,482.7  ±133.1 | 55 | 9,698.8  ±134.0 | 53 | 9,273.8  ±130.3 | 55 | 9,531.4  ±134.8 | 52 | 9,892.7  ±147.3 | 46 |
| 9527 | 8,070.4  ±116.2 | 55 | 8,241.9  ±116.6 | 53 | 7,916.8  ±114.5 | 55 | 8,109.0  ±117.4 | 52 | 8,415.1  ±128.8 | 46 |
| 11454 | 6,912.5  ±103.0 | 55 | 7,045.6  ±102.8 | 53 | 6,806.8  ±102.3 | 55 | 6,941.6  ±103.8 | 52 | 7,203.6  ±114.4 | 46 |
| 13771 | 5,965.4  ±92.8 | 55 | 6,064.4  ±92.2 | 53 | 5,901.1  ±93.1 | 55 | 5,984.8  ±93.2 | 52 | 6,213.2  ±103.4 | 46 |
| 16557 | 5,200.0  ±84.9 | 55 | 5,268.6  ±83.7 | 53 | 5,171.3  ±85.8 | 55 | 5,210.9  ±85.0 | 52 | 5,410.4  ±94.9 | 46 |
| 19905 | 4,587.5  ±78.7 | 55 | 4,630.0  ±77.2 | 53 | 4,589.9  ±80.4 | 55 | 4,590.4  ±78.5 | 52 | 4,767.5  ±88.5 | 46 |
| 23931 | 4,103.4  ±74.4 | 55 | 4,122.6  ±72.5 | 53 | 4,131.8  ±76.6 | 55 | 4,098.8  ±73.9 | 52 | 4,258.9  ±83.9 | 46 |
| 28772 | 3,723.7  ±71.4 | 55 | 3,724.0  ±69.2 | 53 | 3,774.0  ±74.0 | 55 | 3,712.8  ±70.6 | 52 | 3,860.0  ±80.8 | 46 |
| 34591 | 3,430.1  ±69.3 | 55 | 3,414.2  ±66.9 | 53 | 3,497.8  ±72.1 | 55 | 3,413.5  ±68.3 | 52 | 3,551.1  ±78.7 | 46 |
| 41588 | 3,204.5  ±67.9 | 55 | 3,175.  8±65.3 | 53 | 3,286.2  ±70.9 | 55 | 3,183.5  ±66.8 | 52 | 3,313.9  ±77.3 | 46 |
| 50000 | 3,032.9  ±66.9 | 55 | 2,994.0  ±64.2 | 53 | 3,125.2  ±70.0 | 55 | 3,008.0  ±65.6 | 52 | 3,133.1  ±76.3 | 46 |
| 60113 | 2,903.5  ±66.1 | 55 | 2,857.0  ±63.3 | 53 | 3,003.1  ±69.2 | 55 | 2,875.7  ±64.7 | 52 | 2,996.8  ±75.6 | 46 |
| 72272 | 2,806.3  ±65.5 | 55 | 2,754.3  ±62.7 | 53 | 2,911.6  ±68.7 | 55 | 2,776.5  ±64.1 | 52 | 2,894.7  ±75.0 | 46 |
| 86890 | 2,732.7  ±65.0 | 55 | 2,677.0  ±62.2 | 53 | 2,841.9  ±68.2 | 55 | 2,701.6  ±63.6 | 52 | 2,817.3  ±74.5 | 46 |
| 104464 | 2,675.1  ±64.6 | 55 | 2,616.5  ±61.8 | 53 | 2,786.8  ±67.8 | 55 | 2,642.9  ±63.2 | 52 | 2,756.4  ±74.1 | 46 |
| 125593 | 2,627.8  ±64.2 | 55 | 2,567.3  ±61.4 | 53 | 2,741.2  ±67.4 | 55 | 2,594.9  ±62.8 | 52 | 2,706.6  ±73.7 | 46 |
| 150997 | 2,590.2  ±63.9 | 55 | 2,528.4  ±61.0 | 53 | 2,704.9  ±67.1 | 55 | 2,556.8  ±62.4 | 52 | 2,667.2  ±73.3 | 46 |
| 181538 | 2,560.3  ±63.6 | 55 | 2,497.4  ±60.7 | 53 | 2,675.9  ±66.8 | 55 | 2,526.4  ±62.1 | 52 | 2,635.7  ±73.0 | 46 |
| 218256 | 2,534.2  ±63.3 | 55 | 2,470.5  ±60.5 | 53 | 2,650.3  ±66.5 | 55 | 2,500.0  ±61.8 | 52 | 2,608.2  ±72.6 | 46 |
| 262402 | 2,512.3  ±63.0 | 55 | 2,448.2  ±60.2 | 53 | 2,628.9  ±66.2 | 55 | 2,478.1  ±61.5 | 52 | 2,585.0  ±72.3 | 46 |
| 315476 | 2,492.3  ±62.7 | 55 | 2,427.8  ±59.9 | 53 | 2,609.0  ±65.9 | 55 | 2,457.8  ±61.3 | 52 | 2,563.9  ±72.0 | 46 |
| 379286 | 2,473.2  ±62.4 | 55 | 2,408.6  ±59.6 | 53 | 2,590.4  ±65.6 | 55 | 2,438.8  ±61.0 | 52 | 2,544.0  ±71.6 | 46 |
| 456001 | 2,453.8  ±62.0 | 55 | 2,389.3  ±59.3 | 53 | 2,570.9  ±65.2 | 55 | 2,419.4  ±60.6 | 52 | 2,523.8  ±71.2 | 46 |
| 548234 | 2,432.  4±61.6 | 55 | 2,368.0  ±58.8 | 53 | 2,549.2  ±64.7 | 55 | 2,398.  3±60.2 | 52 | 2,501.7  ±70.7 | 46 |
| 659122 | 2,406.8  ±61.0 | 55 | 2,342.7  ±58.3 | 53 | 2,523.2  ±64.1 | 55 | 2,373.0  ±59.6 | 52 | 2,475.2  ±70.1 | 46 |
| 792439 | 2,374.8  ±60.3 | 55 | 2,311.2  ±57.5 | 53 | 2,490.4  ±63.3 | 55 | 2,341.2  ±58.9 | 52 | 2,441.9  ±69.2 | 46 |
| 952721 | 2,332.9  ±59.3 | 55 | 2,269.9  ±56.5 | 53 | 2,447.0  ±62.3 | 55 | 2,299.7  ±57.9 | 52 | 2,398.7  ±68.0 | 46 |
| 1145422 | 2,275.7  ±57.8 | 55 | 2,213.6  ±55.1 | 53 | 2,387.4  ±60.7 | 55 | 2,243.1  ±56.5 | 52 | 2,339.7  ±66.3 | 46 |
| 1377100 | 2,196.2  ±55.8 | 55 | 2,135.7  ±53.2 | 53 | 2,304.8  ±58.7 | 55 | 2,164.3  ±54.5 | 52 | 2,257.9  ±64.1 | 46 |
| 1655638 | 2,086.3  ±53.0 | 55 | 2,027.8±  50.5 | 53 | 2,190.0  ±55.7 | 55 | 2,055.5  ±51.8 | 52 | 2,144.7  ±60.9 | 46 |
| 1990514 | 1,937.1  ±49.2 | 55 | 1,881.8  ±46.9 | 53 | 2,033.7  ±51.8 | 55 | 1,907.4  ±48.1 | 52 | 1,990.6  ±56.6 | 46 |
| 2393124 | 1,744.1  ±44.3 | 55 | 1,693.0  ±42.2 | 53 | 1,831.2  ±46.7 | 55 | 1,716.2  ±43.3 | 52 | 1,791.4  ±51.0 | 46 |
| 2877167 | 1,528.9  ±38.9 | 55 | 1,483.3  ±37.1 | 53 | 1,605.9  ±41.1 | 55 | 1,503.8  ±38.1 | 52 | 1,569.4  ±44.9 | 46 |
| 3459115 | 1,404.1  ±36.2 | 55 | 1,365.4  ±34.8 | 53 | 1,478.9  ±38.5 | 55 | 1,384.3  ±35.7 | 52 | 1,441.4  ±42.0 | 46 |
| 4158770 | 1,662.4  ±44.2 | 55 | 1,627.6  ±43.0 | 53 | 1,763.8  ±47.7 | 55 | 1,651.9  ±44.2 | 52 | 1,711.4  ±51.7 | 46 |
| 4999940 | 2,550.3  ±69.8 | 55 | 2,505.0  ±68.5 | 53 | 2,719.6  ±76.1 | 55 | 2,546.9  ±70.1 | 52 | 2,632.2  ±82.3 | 46 |

Table S3. Statistical analysis for impedance of 5 minutes (Figure S3b)

|  | 1 min | | 2 min | | 3 min | | 4 min | | 5 min | |
| --- | --- | --- | --- | --- | --- | --- | --- | --- | --- | --- |
| Frequency (Hz) | Mean  ±sem | N | Mean  ±sem | N | Mean  ±sem | N | Mean  ±sem | N | Mean  ±sem | N |
| 500 | 171,847.5  ±2,284.6 | 58 | 171,573.4  ±2,313.6 | 58 | 171,584.9  ±2,328.1 | 58 | 171,265.7  ±2,293.2 | 58 | 171,337.3  ±2,305.6 | 58 |
| 601 | 143,993.6  ±1,885.7 | 58 | 144,031.0  ±1,941.3 | 58 | 143,983.0  ±1,946.3 | 58 | 143,840.5  ±1,941.5 | 58 | 143,858.1  ±1,949.5 | 58 |
| 723 | 120,552.3  ±1,599.1 | 58 | 120,630.2  ±1,618.0 | 58 | 120,450.3  ±1,631.5 | 58 | 120,383.4  ±1,610.1 | 58 | 120,240.5  ±1,624.4 | 58 |
| 869 | 102,815.5  ±1,351.0 | 58 | 102,851.2  ±1,382.0 | 58 | 102,759.0  ±1,381.5 | 58 | 102,639.9  ±1,382.2 | 58 | 102,604.3  ±1,378.1 | 58 |
| 1045 | 84,675.5  ±1,107.1 | 58 | 84,791.1  ±1,143.0 | 58 | 84,688.9  ±1,134.7 | 58 | 84,553.0  ±1,137.2 | 58 | 84,536.2  ±1,128.5 | 58 |
| 1256 | 71,693.4  ±937.0 | 58 | 71,742.8  ±964.0 | 58 | 71,675.7  ±962.6 | 58 | 71,571.4  ±958.9 | 58 | 71,517.4  ±955.9 | 58 |
| 1510 | 59,703.7  ±786.1 | 58 | 59,736.0  ±806.2 | 58 | 59,670.1  ±801.0 | 58 | 59,599.6  ±801.4 | 58 | 59,556.5  ±795.0 | 58 |
| 1815 | 50,084.7  ±657.6 | 58 | 50,115.8  ±673.5 | 58 | 50,044.0  ±672.7 | 58 | 49,989.9  ±671.4 | 58 | 49,966.5  ±668.5 | 58 |
| 2183 | 42,029.4  ±553.6 | 58 | 42,055.5  ±567.6 | 58 | 42,001.3  ±564.6 | 58 | 41,959.1  ±565.1 | 58 | 41,931.8  ±562.0 | 58 |
| 2624 | 35,301.7  ±465.9 | 58 | 35,319.1  ±477.1 | 58 | 35,265.1  ±475.1 | 58 | 35,228.8  ±474.4 | 58 | 35,210.2  ±473.0 | 58 |
| 3155 | 29,651.6  ±392.2 | 58 | 29,646.2  ±400.7 | 58 | 29,610.5  ±398.5 | 58 | 29,570.3  ±398.6 | 58 | 29,564.4  ±397.8 | 58 |
| 3793 | 24,943.4  ±331.3 | 58 | 24,927.7  ±337.6 | 58 | 24,905.0  ±336.8 | 58 | 24,872.2  ±336.4 | 58 | 24,862.1  ±335.8 | 58 |
| 4560 | 20,979.6  ±280.3 | 58 | 20,962.5  ±285.2 | 58 | 20,941.0  ±284.9 | 58 | 20,915.7  ±284.2 | 58 | 20,909.2  ±284.0 | 58 |
| 5482 | 17,693.0  ±238.1 | 58 | 17,666.1  ±241.8 | 58 | 17,648.1  ±241.9 | 58 | 17,626.6  ±241.2 | 58 | 17,626.1  ±241.2 | 58 |
| 6591 | 14,944.1  ±203.5 | 58 | 14,906.6  ±206.0 | 58 | 14,892.8  ±206.1 | 58 | 14,876.7  ±205.6 | 58 | 14,878.0  ±205.5 | 58 |
| 7924 | 12,652.4  ±174.8 | 58 | 12,606.3  ±176.2 | 58 | 12,595.7  ±176.2 | 58 | 12,585.0  ±175.9 | 58 | 12,589.2  ±176.1 | 58 |
| 9527 | 10,752.3  ±151.1 | 58 | 10,695.4  ±151.8 | 58 | 10,687.3  ±151.7 | 58 | 10,681.7  ±151.8 | 58 | 10,688.7  ±151.8 | 58 |
| 11454 | 9,185.1  ±132.1 | 58 | 9,116.8  ±131.8 | 58 | 9,112.3  ±131.9 | 58 | 9,109.6  ±131.8 | 58 | 9,121.0  ±132.2 | 58 |
| 13771 | 7,893.7  ±116.8 | 58 | 7,814.6  ±115.7 | 58 | 7,812.8  ±116.0 | 58 | 7,813.8  ±116.0 | 58 | 7,828.0  ±116.4 | 58 |
| 16556 | 6,836.6  ±104.9 | 58 | 6,745.8  ±103.2 | 58 | 6,746.4  ±103.4 | 58 | 6,751.1  ±103.6 | 58 | 6,768.6  ±104.2 | 58 |
| 19905 | 5,977.8  ±95.9 | 58 | 5,873.8  ±93.5 | 58 | 5,876.9  ±93.8 | 58 | 5,885.1  ±94.1 | 58 | 5,905.4  ±94.6 | 58 |
| 23931 | 5,286.8  ±88.8 | 58 | 5,170.6  ±85.9 | 58 | 5,176.0  ±86.2 | 58 | 5,187.5  ±86.6 | 58 | 5,211.0  ±87.3 | 58 |
| 28772 | 4,736.6  ±83.5 | 58 | 4,608.6  ±80.1 | 58 | 4,615.9  ±80.4 | 58 | 4,630.3  ±80.8 | 58 | 4,656.9  ±81.7 | 58 |
| 34591 | 4,302.5  ±79.6 | 58 | 4,163.7  ±75.9 | 58 | 4,173.2  ±76.1 | 58 | 4,190.3  ±76.7 | 58 | 4,219.1  ±77.5 | 58 |
| 41588 | 3,963.9  ±76.8 | 58 | 3,815.4  ±72.9 | 58 | 3,826.2  ±73.2 | 58 | 3,845.8  ±73.7 | 58 | 3,877.4  ±74.6 | 58 |
| 50000 | 3,701.9  ±74.8 | 58 | 3,545.5  ±70.6 | 58 | 3,558.0  ±71.0 | 58 | 3,579.3  ±71.6 | 58 | 3,613.0  ±72.5 | 58 |
| 60113 | 3,502.3  ±73.4 | 58 | 3,339.0  ±69.1 | 58 | 3,352.5  ±69.5 | 58 | 3,375.7  ±70.1 | 58 | 3,410.7  ±71.0 | 58 |
| 72272 | 3,350.7  ±72.4 | 58 | 3,182.4  ±68.0 | 58 | 3,197.0  ±68.4 | 58 | 3,221.5  ±69.0 | 58 | 3,257.9  ±70.0 | 58 |
| 86889 | 3,235.8  ±71.6 | 58 | 3,063.7  ±67.2 | 58 | 3,079.2  ±67.6 | 58 | 3,104.6  ±68.2 | 58 | 3,141.9  ±69.2 | 58 |
| 104464 | 3,146.1  ±70.9 | 58 | 2,971.5  ±66.5 | 58 | 2,987.2  ±66.9 | 58 | 3,013.5  ±67.5 | 58 | 3,051.5  ±68.4 | 58 |
| 125593 | 3,073.8  ±70.3 | 58 | 2,897.4  ±65.9 | 58 | 2,913.6  ±66.3 | 58 | 2,940.4  ±66.9 | 58 | 2,978.8  ±67.8 | 58 |
| 150997 | 3,017.5  ±69.8 | 58 | 2,839.9  ±65.4 | 58 | 2,856.4  ±65.7 | 58 | 2,883.6  ±66.4 | 58 | 2,922.3  ±67.3 | 58 |
| 181538 | 2,973.2  ±69.3 | 58 | 2,795.1  ±64.9 | 58 | 2,811.9  ±65.3 | 58 | 2,839.3  ±65.9 | 58 | 2,878.1  ±66.9 | 58 |
| 218256 | 2,935.6  ±68.9 | 58 | 2,757.1  ±64.5 | 58 | 2,774.0  ±64.9 | 58 | 2,801.5  ±65.5 | 58 | 2,840.7  ±66.4 | 58 |
| 262402 | 2,905.0  ±68.6 | 58 | 2,726.2  ±64.1 | 58 | 2,743.1  ±64.5 | 58 | 2,770.9  ±65.1 | 58 | 2,810.0  ±66.1 | 58 |
| 315476 | 2,877.4  ±68.2 | 58 | 2,698.7  ±63.7 | 58 | 2,715.8  ±64.1 | 58 | 2,743.6  ±64.7 | 58 | 2,783.0  ±65.7 | 58 |
| 379286 | 2,852.0  ±67.8 | 58 | 2,673.4  ±63.4 | 58 | 2,690.6  ±63.7 | 58 | 2,718.5  ±64.4 | 58 | 2,757.6  ±65.3 | 58 |
| 456001 | 2,826.7  ±67.4 | 58 | 2,648.5  ±62.9 | 58 | 2,665.8  ±63.3 | 58 | 2,693.6  ±63.9 | 58 | 2,733.0  ±64.9 | 58 |
| 548234 | 2,799.5  ±66.9 | 58 | 2,622.0  ±62.4 | 58 | 2,639.2  ±62.8 | 58 | 2,667.0  ±63.4 | 58 | 2,706.3  ±64.4 | 58 |
| 659122 | 2,767.7  ±66.2 | 58 | 2,591.3  ±61.8 | 58 | 2,608.5  ±62.2 | 58 | 2,636.2  ±62.8 | 58 | 2,675.3  ±63.8 | 58 |
| 792439 | 2,729.0  ±65.4 | 58 | 2,554.0  ±61.0 | 58 | 2,571.1  ±61.4 | 58 | 2,598.7  ±62.0 | 58 | 2,637.6  ±63.0 | 58 |
| 952721 | 2,678.9  ±64.3 | 58 | 2,506.2  ±60.0 | 58 | 2,523.3  ±60.3 | 58 | 2,550.5  ±61.0 | 58 | 2,589.0  ±61.9 | 58 |
| 1145422 | 2,612.0  ±62.8 | 58 | 2,442.3  ±58.5 | 58 | 2,459.1  ±58.9 | 58 | 2,485.9  ±59.5 | 58 | 2,524.0  ±60.5 | 58 |
| 1377100 | 2,520.2  ±60.7 | 58 | 2,355.2  ±56.5 | 58 | 2,371.6  ±56.9 | 58 | 2,397.6  ±57.5 | 58 | 2,434.8  ±58.4 | 58 |
| 1655638 | 2,394.4  ±57.8 | 58 | 2,235.7  ±53.7 | 58 | 2,251.3  ±54.0 | 58 | 2,276.6  ±54.6 | 58 | 2,312.5  ±55.6 | 58 |
| 1990514 | 2,224.0  ±53.8 | 58 | 2,074.1  ±49.9 | 58 | 2,089.0  ±50.3 | 58 | 2,113.0  ±50.8 | 58 | 2,146.9  ±51.7 | 58 |
| 2393124 | 2,003.2  ±48.7 | 58 | 1,866.0  ±45.1 | 58 | 1,879.7  ±45.4 | 58 | 1,901.5  ±45.9 | 58 | 1,932.9  ±46.8 | 58 |
| 2877167 | 1,757.9  ±43.2 | 58 | 1,635.2  ±39.9 | 58 | 1,647.2  ±40.1 | 58 | 1,667.0  ±40.6 | 58 | 1,695.1  ±41.4 | 58 |
| 3459115 | 1,618.3  ±40.7 | 58 | 1,504.0  ±37.5 | 58 | 1,515.4  ±37.7 | 58 | 1,533.7  ±38.2 | 58 | 1,559.9  ±39.0 | 58 |
| 4158770 | 1,924.2  ±50.6 | 58 | 1,790.3  ±46.4 | 58 | 1,803.5  ±46.8 | 58 | 1,825.2  ±47.4 | 58 | 1,855.6  ±48.3 | 58 |
| 4999940 | 2,960.8  ±81.2 | 58 | 2,756.6  ±74.3 | 58 | 2,777.1  ±74.9 | 58 | 2,810.0  ±75.9 | 58 | 2,856.3  ±77.4 | 58 |

Table S4. Statistical analysis for impedance of 3 eDMAs (Figure S3c)

|  | eDMA 5958 | | eDMA 5959 | | eDMA 5953 | |
| --- | --- | --- | --- | --- | --- | --- |
| Frequency (Hz) | mean±sem | N | mean±sem | N | mean±sem | N |
| 500 | 171,903.7±2,316.2 | 58 | 132,733.9±1,753.9 | 55 | 130,939.7±1,608.6 | 57 |
| 601 | 144,357.3±1,948.8 | 58 | 111,259.6±1,469.2 | 55 | 109,861.1±1,344.4 | 57 |
| 723 | 120,688.1±1,623.9 | 58 | 92,853.8±1,226.4 | 55 | 91,733.7±1,122.1 | 57 |
| 869 | 103,018.1±1,388.1 | 58 | 79,047.9±1,050.2 | 55 | 78,182.9±960.7 | 57 |
| 1045 | 84,844.4±1,139.4 | 58 | 64,993.5±856.1 | 55 | 64,278.9±783.2 | 57 |
| 1256 | 71,871.6±966.1 | 58 | 54,953.1±725.5 | 55 | 54,426.5±666.7 | 57 |
| 1510 | 59,825.2±803.5 | 58 | 45,649.0±602.5 | 55 | 45,237.3±552.7 | 57 |
| 1815 | 50,168.4±673.3 | 58 | 38,222.1±505.5 | 55 | 37,921.0±465.0 | 57 |
| 2183 | 42,092.0±565.7 | 58 | 32,028.3±424.3 | 55 | 31,815.1±390.6 | 57 |
| 2624 | 35,345.1±475.7 | 58 | 26,852.7±356.8 | 55 | 26,700.7±328.7 | 57 |
| 3155 | 29,666.1±400.1 | 58 | 22,511.9±300.4 | 55 | 22,421.3±277.4 | 57 |
| 3793 | 24,944.2±337.2 | 58 | 18,912.5±254.0 | 55 | 18,862.8±234.8 | 57 |
| 4560 | 20,971.4±284.8 | 58 | 15,889.7±215.2 | 55 | 15,870.1±199.1 | 57 |
| 5482 | 17,669.5±241.6 | 58 | 13,387.0±183.4 | 55 | 13,390.7±169.9 | 57 |
| 6591 | 14,907.0±205.5 | 58 | 11,303.0±157.3 | 55 | 11,322.7±145.7 | 57 |
| 7924 | 12,604.9±175.9 | 58 | 9,575.9±135.9 | 55 | 9,607.1±125.8 | 57 |
| 9527 | 10,691.7±151.5 | 58 | 8,150.6±118.7 | 55 | 8,187.9±109.8 | 57 |
| 11454 | 9,111.9±131.5 | 58 | 6,982.0±105.3 | 55 | 7,021.4±97.2 | 57 |
| 13771 | 7,808.3±115.6 | 58 | 6,025.8±94.9 | 55 | 6,063.7±87.4 | 57 |
| 16556 | 6,738.8±103.1 | 58 | 5,252.2±86.9 | 55 | 5,285.9±79.7 | 57 |
| 19905 | 5,866.4±93.5 | 58 | 4,633.1±80.7 | 55 | 4,660.3±73.7 | 57 |
| 23931 | 5,162.8±85.9 | 58 | 4,143.1±76.3 | 55 | 4,162.8±69.4 | 57 |
| 28772 | 4,600.5±80.1 | 58 | 3,758.9±73.2 | 55 | 3,771.0±66.4 | 57 |
| 34591 | 4,155.5±76.0 | 58 | 3,461.3±71.1 | 55 | 3,465.8±64.3 | 57 |
| 41588 | 3,807.4±73.0 | 58 | 3,232.8±69.6 | 55 | 3,230.1±62.8 | 57 |
| 50000 | 3,537.7±70.9 | 58 | 3,058.6±68.6 | 55 | 3,050.4±61.7 | 57 |
| 60113 | 3,331.5±69.4 | 58 | 2,927.2±67.8 | 55 | 2,914.5±61.0 | 57 |
| 72272 | 3,175.4±68.3 | 58 | 2,828.7±67.2 | 55 | 2,812.9±60.5 | 57 |
| 86889 | 3,057.2±67.5 | 58 | 2,754.1±66.7 | 55 | 2,736.5±60.0 | 57 |
| 104464 | 2,965.2±66.8 | 58 | 2,695.6±66.3 | 55 | 2,677.1±59.6 | 57 |
| 125593 | 2,891.4±66.2 | 58 | 2,647.5±65.9 | 55 | 2,629.2±59.3 | 57 |
| 150997 | 2,834.3±65.7 | 58 | 2,609.5±65.5 | 55 | 2,591.8±59.0 | 57 |
| 181538 | 2,789.7±65.2 | 58 | 2,579.1±65.2 | 55 | 2,562.3±58.8 | 57 |
| 218256 | 2,751.9±64.8 | 58 | 2,552.6±64.9 | 55 | 2,537.1±58.6 | 57 |
| 262402 | 2,721.3±64.4 | 58 | 2,530.5±64.6 | 55 | 2,516.2±58.3 | 57 |
| 315476 | 2,694.1±64.1 | 58 | 2,510.2±64.3 | 55 | 2,497.1±58.1 | 57 |
| 379286 | 2,669.0±63.7 | 58 | 2,491.0±64.0 | 55 | 2,479.3±57.9 | 57 |
| 456001 | 2,644.3±63.2 | 58 | 2,471.5±63.7 | 55 | 2,460.8±57.6 | 57 |
| 548234 | 2,618.2±62.7 | 58 | 2,450.0±63.2 | 55 | 2,440.4±57.2 | 57 |
| 659122 | 2,587.7±62.1 | 58 | 2,424.2±62.6 | 55 | 2,415.5±56.7 | 57 |
| 792439 | 2,550.7±61.3 | 58 | 2,391.9±61.8 | 55 | 2,384.0±56.0 | 57 |
| 952721 | 2,503.2±60.2 | 58 | 2,349.6±60.8 | 55 | 2,342.5±55.0 | 57 |
| 1145422 | 2,439.6±58.8 | 58 | 2,291.9±59.3 | 55 | 2,285.6±53.7 | 57 |
| 1377100 | 2,352.6±56.7 | 58 | 2,211.8±57.2 | 55 | 2,206.5±51.9 | 57 |
| 1655638 | 2,233.4±53.9 | 58 | 2,100.9±54.4 | 55 | 2,096.6±49.4 | 57 |
| 1990514 | 2,072.0±50.1 | 58 | 1,950.1±50.5 | 55 | 1,947.3±45.9 | 57 |
| 2393124 | 1,864.1±45.3 | 58 | 1,755.2±45.5 | 55 | 1,753.9±41.4 | 57 |
| 2877167 | 1,633.3±39.9 | 58 | 1,538.3±40.0 | 55 | 1,538.5±36.5 | 57 |
| 3459115 | 1,501.8±37.5 | 58 | 1,414.8±37.4 | 55 | 1,414.4±34.1 | 57 |
| 4158770 | 1,786.3±46.4 | 58 | 1,683.4±46.2 | 55 | 1,674.9±41.7 | 57 |
| 4999940 | 2,749.1±74.1 | 58 | 2,590.8±73.4 | 55 | 2,563.8±65.8 | 57 |

**Reference**

[1] M. Benz, M.R. Molla, A. Boser, A. Rosenfeld, and P.A. Levkin, *Nat Commun*, **2019**, *10*,2879.
